# Comparative Ecology of Capsular *Exophiala* Species Causing Disseminated Infection in Humans

**DOI:** 10.3389/fmicb.2017.02514

**Published:** 2017-12-19

**Authors:** Yinggai Song, Wendy W. J. Laureijssen-van de Sande, Leandro F. Moreno, Bert Gerrits van den Ende, Ruoyu Li, Sybren de Hoog

**Affiliations:** ^1^Department of Dermatology, Peking University First Hospital, Beijing, China; ^2^Research Center for Medical Mycology, Peking University, Beijing, China; ^3^Beijing Key Laboratory of Molecular Diagnosis of Dermatoses, Peking University First Hospital, Beijing, China; ^4^Westerdijk Fungal Biodiversity Institute, Utrecht, Netherlands; ^5^Department of Medical Microbiology and Infectious Diseases, Erasmus MC, University of Rotterdam, Rotterdam, Netherlands; ^6^Center of Expertise in Mycology Radboudumc/CWZ, Radboud University Nijmegen Medical Center, Nijmegen, Netherlands

**Keywords:** black yeast, capsule, *Exophiala* species, virulence, pathogenicity, opportunism, physiology, *Galleria mellonella*

## Abstract

*Exophiala spinifera* and *Exophiala dermatitidis* (Fungi: *Chaetothyriales*) are black yeast agents potentially causing disseminated infection in apparently healthy humans. They are the only *Exophiala* species producing extracellular polysaccharides around yeast cells. In order to gain understanding of eventual differences in intrinsic virulence of the species, their clinical profiles were compared and found to be different, suggesting pathogenic strategies rather than coincidental opportunism. Ecologically relevant factors were compared in a model set of strains of both species, and significant differences were found in clinical and environmental preferences, but virulence, tested in *Galleria mellonella* larvae, yielded nearly identical results. Virulence factors, i.e., melanin, capsule and muriform cells responded in opposite direction under hydrogen peroxide and temperature stress and thus were inconsistent with their hypothesized role in survival of phagocytosis. On the basis of physiological profiles, possible natural habitats of both species were extrapolated, which proved to be environmental rather than animal-associated. Using comparative genomic analyses we found differences in gene content related to lipid metabolism, cell wall modification and polysaccharide capsule production. Despite the fact that both species cause disseminated infections in apparently healthy humans, it is concluded that they are opportunists rather than pathogens.

## Introduction

The black yeast genus *Exophiala* (Fungi, order *Chaetothyriales*) contains about 40 species, 17 of which have been reported from human infections (De Hoog et al., 2000b; Revankar and Sutton, 2010). Some species are notorious agents of deep and disseminated human infection, in debilitated but also in healthy individuals (Revankar et al., 2002). In contrast to frequent statements in the literature, *Exophiala* black yeasts are not common saprobes on plant debris but are selected by domesticated environments where conditions for microbial growth are relatively hostile. Their survival strategy has been referred to as polyextremotolerant (Gostincar et al., 2011).

Of all *Exophiala* species, *Exophiala dermatitidis* (*E. dermatitidis*) and *Exophiala spinifera* (*E. spinifera*) are associated with the most severe infections, which in systemic cases have high mortality rates of up to 80% (Rajam et al., 1958; Crosby et al., 1989; Campos-Takaki and Jardim, 1994; de Hoog et al., 2005; Radhakrishnan et al., 2010; Li et al., 2011; Patel et al., 2013; Hu et al., 2014; Wang et al., 2015; Chen et al., 2016). As a possible explanation of their relatively high virulence compared to other *Exophiala* species, the occurrence of extracellular polysaccharide on yeast cells has been mentioned, masking the cells for human phagocytes upon tissue invasion (Yurlova and de Hoog, 2002). *Exophiala dermatitidis* has a global distribution in the domesticated environment, but cases of deep phaeohyphomycosis are nearly exclusively found in East Asia (Revankar et al., 2002; Kantarcioglu et al., 2004). In Europe the fungus occurs as a respiratory colonizer in patients with cystic fibrosis (Kondori et al., 2011). In contrast to many other opportunistic fungi its frequency seems to be relatively unaffected by the growing hospitalized populations of patients with compromised immunity. Extended searches for the fungus in the natural environment yielded feces of frugivorous tropical animals as a possible niche, while prevalence in soil and plant debris was close to zero (Sudhadham et al., 2008). The species is however commonly found in indoor wet cells such as bathing facilities and dishwashers (Matos et al., 2002; Gümral et al., 2015) and other human-made environments such as creosoted railway sleepers (Gumral et al., 2014). These habitats are characterized by (i) high temperatures, (ii) osmotic stress, (iii) acidic or alkaline conditions, and (iv) toxicity along with (v) low nutrient availability. It has been speculated that such strongly selective environments may drive their evolution toward human pathogenicity (Gostincar et al., 2011; Dogen et al., 2013b; Zupancic et al., 2016).

*Exophiala spinifera* is rare, both in humans and in the environment. Disseminated infections may have a fatal outcome and were prevalently observed in immunocompetent children and adolescents, while in the elderly infections tend to remain as (sub) cutaneous lesions, taking a mild course despite underlying disorders (de Hoog et al., 1999). The species has not been reported from CF lungs. Its environmental occurrence displays a rather scattered picture.

The ecological differences between *E. dermatitidis* and *E. spinifera* are intriguing. Both are characterized by the production of extracellular slimes, which may be either in the form of a well-delimited capsule or of diffusely exuded exopolysaccharides (EPS). The capsular material was reported around very young cells of *E. spinifera* and acid mucopolysaccharides were observed around yeast cells of *E. dermatitidis* (Yurlova and de Hoog, 2002). In general, capsular material is a key determinant of virulence, as extracellular polysaccharides have a significant role in adherence, impairment of phagocytosis and to reduce complement-mediated killing (Nishimura and Miyaji, 1983). If the two species are opportunists without pathogenic strategies, the average clinical course of both is expected to be similar, i.e., dependent on host conditions and route of infection. Alternatively, the striking differences between the two species have to be explained by their environmental behavior. In the present study our systematic approach involves growth, morphology of invasive phases, multilocus sequencing, and physiology, while relative virulence was determined in a *Galleria mellonella* larvae model. In addition, we assessed the genomes of *E. spinifera* and *E. dermatitidis* in order to provide gene information on the physiological variations observed between the species.

## Materials and methods

### Literature search

Keywords “*Exophiala spinifera*,” “*Exophiala dermatitidis*,” and “*Wangiella dermatitidis*” were used in PubMed to search for English literature including research articles, reviews and case reports published until January 2017. In addition, research databases available at Westerdijk Institute were consulted.

### Strains studied

A global set of 48 *E. spinifera* isolates (26 clinical, 22 environmental) and 47 *E. dermatitidis* isolates (28 clinical, 19 environmental) were available for study (Table 1). Strains were obtained from the Research Center for Medical Mycology at Peking University and the Centraalbureau voor Schimmelcultures (housed at Westerdijk Fungal Biodiversity Institute, Utrecht, The Netherlands) from 1997 to 2016. Representatives of genotypes A1, A2, A3, genotype B, and genotype C of *E. dermatitidis* were included (Table 1). Data of prevalence of the two species were abstracted from a research database on black yeasts at Westerdijk Institute, comprising ITS and part of *TEF1* sequenced items of *E. spinifera* and *E. dermatitidis*, respectively.

**Table 1:** Strains analyzed of *E. spinifera* and *E. dermatitidis*.

**Accession number**	**Country**	**Source**	**Physiology**	***Galleria mellonella***	**GenBank accession No**.		
					**ITS**	***TEF1***	***BT2***
CBS 131564	Thailand	Patient	x	x	MF039709	MF067279	MF067274
CBS 101543	USA	Patient	x				
CBS 101545	USA	Patient					
CBS 102179	Senegal	Patient	x		KP132127.1	MF067282	MF067269
CBS 119098	USA	Elbow lesion	x		KP132128.1		KF928553.1
BMU00048	China	Face	x	x	EU910266.1		
BMU00049	China	Face	x	x	EU910257.1		
CBS 125607	India	Arm	x	x	GU980971.1		
CBS 129971	China	Patient	x				
CBS 194.61	India	Patient	x		AY156958.1	MF067284	
CBS 269.28	India	Skin	x		AY156960.1	MF067280	MF067272
CBS 899.68	USA	Face	x	x	NR_111131.1	EF551541.1	EF551516.1
CBS 102460	India	Patient					
BMU00221	China	Human			MF039707	MF067276	MF067271
CBS 101533	China	Bark	x	x	AY156971.1		
CBS 101534	China	Soil			MF039706	MF067275	
CBS 101535	China	Soil					
CBS 101537	Venezuela	Rotten cactus			AY156970.1	MF067286	
CBS101538	China	Bark			JX473274.1	EF551523.1	EF551506.1
CBS 101539	Colombia	Soil	x	x	AY156969.1	MF067278	MF067268
CBS 101542	Colombia	Soil			AY156967.1	EF551527.1	EF551498.1
CBS 101644	USA	Mouldy maize kernel			EF551460.1	EF551543.1	EF551513.1
CBS 110628	Venezuela	Bark			AY156966.2		
CBS 116557	Thailand	Pine apple	x	x	MF039710		
CBS 118937	Papua New Guinea	Sago starch					MF067270
CBS 236.93	Germany	Apple juice			AY156959.1	MF067285	
CBS 425.92	Germany	Apple juice	x		EF551459.1	EF551539.1	EF551515.1
CBS 667.76	Uruguay	Butiayatay		x	AY156964.1		
CBS 670.76	Uruguay	Nest of Anumbis anumbi	x		AF549451.1		
CBS 671.76	Uruguay	Nest of Anumbis anumbi			AY156975.1		
CBS 126013	Brazil	Babassu coconut shell	x	x			
CBS 126734	Brazil	Babassu coconut shell					
CBS 126862	Brazil	Babassu coconut shell					MF067273
CBS 127023	Brazil	Horse manure	x		MF039708	MF067277	
CBS 126730	Brazil	Babassu palm tree			MF039711	MF067281	MF067267
CBS 126726	Brazil	Rotting wood	x		MF039712	MF067283	MF067266
UTHSC 91-188	USA	Patient			EF025417.1		
UTHSC R-2959	USA	Patient					
UTHSC R-773	USA	Patient					
UTHSC 88-15	USA	Patient					
UTHSC R-1443	USA	Patient					
UTHSC 97-2073	USA	Patient					
UTHSC R-2870	USA	Patient					
UTHSC R-2955	USA	Patient					
BMU08022	China	Patient					
CBS 356.83	Egypt	Patient					
CBS 132590	India	Patient			MF039713		
CBS 101544	USA	Scalp					
CBS 116726	Thailand	Railway				MF320204	
CBS 134010	Netherlands	Turkish sauna	x				
CBS 120483	Thailand	Railway	x	x	MF320170		
CBS 552.90	Germany	Patient	x		MF320158	MF320192	MF320214
CBS 525.76	Japan	Patient	x				
CBS 292.49	USA	Stool	x		AY554286.1	MF320181	MF320216
CBS 207.35	Japan	Skin	x	x	KF928444.1	MF320177	KF928572.1
CBS 578.76	Japan	Brain	x		MF320154	MF320193	MF320213
CBS 581.76	Japan	Brain				MF320190	MF320208
CBS 338.90	Germany	Patient				MF320191	
CBS 120443	Thailand	Steam bath	x	x		MF320195	MF320215
CBS 109154	South Korea	Brain	x	x	AY857525.1	MF320194	MF320219
CBS 120550	Austria	Steam bath	x	x	MF320147	MF320197	MF320218
CBS 686.92	Germany	Blood	x	x	MF320155	MF320184	MF320209
CBS 123474	Turkey	Neck	x				
CBS 115663	Qatar	Respiratory	x	x	AY663828.1	MF320198	
CBS 120429	Finland	Patient	x		MF320151	MF320178	MF320211
CBS 100340	Germany	Patient					MF320223
CBS 150.90	Germany	Patient					MF320225
CBS 120473	USA	Brain	x	x	MF320159	MF320196	MF320217
CBS 120472	USA	Leg lesion	x		MF320163		
CBS 109144	Netherlands	Steam bath	x		MF320156	MF320183	
CBS 151.93	Germany	Root Tilia			MF320167	MF320206	
CBS 120479	Thailand	Railway				MF320188	
CBS 154.90	Germany	Patient				MF320186	
CBS 736.87	Ireland	Beer			MF320173	MF320203	MF320221
CBS 552.92	Germany	Patient			MF320169	MF320207	
CBS 109149	Slovenia	Bath tube	x		MF320161		
CBS 109136	Netherlands	Steam bath			MF320162	MF320201	MF320210
CBS 109140	Finland	Steam bath			MF320146	MF320180	MF320212
CBS 109143	Netherlands	Sauna			MF320152	MF320185	
CBS 577.76	Japan	Brain	x			MF320182	
CBS 100339	Germany	Patient			MF320153	MF320205	
CBS 109142	Netherlands	Berry			MF320160	MF320202	MF320222
CBS 100341	Germany	Patient					
CBS 109148	Netherlands	Human feces			MF320157	MF320179	MF320220
CBS 132754	Turkey	Bath tube	x	x	MF320176	MF320199	
CBS 132758	Turkey	Dishwasher	x	x	MF320171	MF320200	
CBS 149.90	Germany	Sputum					MF320224
CBS 971.87	Unknown	Patient			MF320164		
BMU00031	China	Patient			MF320145		
BMU00032	China	Patient			MF320148		
CBS 424.67	Germany	Skin			MF320149		
CBS 109145	Netherlands	Steambath			MF320150		
CBS 156.90	Germany	Cystic fibrosis			MF320175		
CBS 120579	Thailand	Steambath			MF320174		
BMU00037	China	Wood					
BMU00044	China	Patient			MF320172		

*x, Strains used in subsequent experiments (physiology and Galleria model)*.

Twenty *E. spinifera* strains and twenty *E. dermatitidis* strains (Table 1), representing maximum ecological and geographical variation, were selected for physiology testing and the *G. mellonella* virulence model. Identity of strains was verified by sequencing.

### DNA extraction

Genomic DNA was obtained from strains grown for 7–14 day on MEA at 24°C. All cultures were handled within a class II biological safety cabinet. Extraction was followed by the cetyltrimethylammonium bromide (CTAB) protocol according to CBS. Quality and quantity of isolated DNA was verified on a NanoDrop ND-1000 Spectrophotometer using ND-1000 v3.3.0 software (Coleman Technologies, Wilmington, DE, USA). Samples were stored at −20°C.

### DNA amplification and sequencing

The following nuclear genes were amplified by PCR: rDNA internal transcribed spacer region (ITS), partial transcriptional elongation factor 1 subunit α (*TEF1*-α), and β-tubulin (*TUB*). ITS of the rDNA operon was amplified with ITS1 and ITS4. Partial β-tubulin (*TUB*), covering the variable 5′-end containing four small introns, was amplified with TUB2a and TUB2b, the partial gene *TEF1*-α with EF1-728F and EF1-986R primer set. Conditions for amplifications of all genes were as described by de Hoog et al. (2011). Sequencing was done with an ABI3730 automatic sequencer (Applied Biosystems, Foster City, CA, USA) and sequence data were adjusted by SeqManPro (DNAStar, Madison, WI, USA). GenBank accession numbers are given in Table 1. We selectively submitted some of the sequences per clade.

### Alignment and phylogenetic reconstruction

For phylogenetic reconstructions with different loci resulting in different degrees of resolution, appropriate reference sequences were obtained from GenBank. Multiple sequence alignments were created with online Mafft v7 using automatic alignment strategy. Alignments were reviewed and corrected manually. Ambiguously aligned regions, long gaps and introns were removed from the alignments using BioEdit v7.1.3.0. Phylogenetic reconstructions were done for each locus using maximum likelihood (ML) implemented in Mega v6.06, and MrBayes trees were done via the Cipres portal (http://www.phylo.org/). Mega v6.06 selected K2+G as the most appropriate model of DNA substitution for ML analysis. Support for the internodes was assessed by bootstrap analysis from 1,000 replicates. Trees were viewed and edited with Mega v6.06, FigTree v1.4.2 and Adobe Illustrator CS6.

### Morphology

Slides were made by Shear's mounting medium without pigments. Micrographs were taken using a Nikon Eclipse 80i microscope and DS Camera Head DS-Fi1/DS-5 m/DS-2Mv/DS-2MBW using NIS-Element freeware package (Nikon Europe, Badhoevedorp, The Netherlands). Dimensions were taken with the Nikon Eclipse 80i measurement module on slides and the mean and standard deviation were calculated from measurements of 40–50 conidia.

Twenty *E. spinifera* and twenty *E. dermatitidis* strains were tested for the production of muriform cells known as the invasive phase of human chromoblastomycosis. Strains were incubated at 25 and 37°C for 1 week in liquid acidic medium (30 g glucose, 3 g NaNO_3_, 0.01 g FeSO_4_·7H_2_O, 0.265 g NH_4_Cl, 0.003 g thiamin, 1 mM CaCl_2_ in 1 L dH_2_O, pH adjusted at 2.5 with HCl) shaken at 150 r.p.m (Karuppayil and Szaniszlo, 1997).

### Capsule

Strains were maintained on Potato Dextrose Agar (PDA) slants, inoculated on fresh PDA plates at 24 and 37°C and incubated for 7 days. Presence of extracellular polysaccharide was verified regularly during 2–7 days of growth and capsular sizes were measured with negative staining in India ink (Yurlova and de Hoog, 2002). All tests were performed three times in duplicate. Numerical values are the means of at least 20 determinations.

### Physiology

All tests were done with 20-selected *E. spinifera* and 20 *E. dermatitidis* strains. Cardinal growth temperatures were determined on 2% malt extract agar (MEA; Difco). Plates were incubated at 15–45°C with 3°C intervals in the dark for 2 weeks; plates contained double quantities of medium and were sealed to prevent drying out. Colony diameters were measured for a selection of 20 *E. spinifera* strains and 20 *E. dermatitidis* strains based on phylogenetic results and references. In addition, growth responses at 40 and 45°C were recorded. To evaluate whether 40 and 45°C were fungicidal, the cultures were returned to 24°C after 2 weeks and incubated for two additional weeks. Experiments consisted of three simultaneous replicates for each isolate; the entire procedure was repeated once.

Lipases were tested with Tween 80 opacity test medium (TOTM) according to Slifkin (Slifkin, 2000), incubating Petri dishes at 24°C for 14 day. Proteolysis was tested with Bromocresol purple-milk solids-glucose agar (BCP-MS-G) medium (Fischer and Kane, 1971; Summerbell et al., 1988) using colony fragments as inoculum. After incubation at 24°C for 14 d, color changes of the medium were recorded. Haemolytic activity was evaluated by culturing isolates on blood agar (BioMérieux, Marcy-l'Étoile, France) for 14 d at 24°C. Positive reaction is a clear ring of hemolysis around the colony. Production of urease was determined in Christensen's urea broth after incubation at 24 and 37°C for 8 and 24 h, with a final check after 7 day. Acid productions was tested on Custer's chalk medium including 5% glucose and 0.5% calcium carbonate after incubation at 25°C for 2 weeks.

Oxygenic stress was evaluated with cultures in YEPD agar medium with hydrogen peroxide to reach concentrations of 3, 6, 9, and 12 mM after sterilization. Growth rates and morphology were recorded. Cycloheximide 0.2% (Sigma-Aldrich, Zwijndrecht, The Netherlands) tolerance was evaluated by growing isolates on SGA with and without cycloheximide incubated at 24 and 37°C for 2 wks. Osmotolerance was tested with YPD basic medium with 20, 40 and 60% sucrose. Halotolerance was tested with complete medium with 2.5, 5, and 10% NaCl and MgCl_2_.

### Protein family classification

For functional annotation of protein sets corresponding to *E. dermatitidis* CBS 525.76 and *E. spinifera* CBS 899.68, sequences were retrieved from the Black Yeast Genome Database (Moreno et al., 2017). Genes correlated with capsule production were predicted by identifying orthologs using the OrthoMCL pipeline, previously described in *Cryptococcus neoformans* (Gish et al., 2016). Lipase family classification was performed based on the top BLAST hit (cut-off *e*-value > 1e^−15^) to the lipase-engineering database (Barth et al., 2004). Genes involved in the nitrogen metabolism were predicted mapping selected urease genes known from previously work (Carlini and Ligabue-Braun, 2016). Catalase/peroxidases genes were predicted via top BLAST hit (cut-off *e*-value > 1e^−15^) to the PeroxiBase (Fawal et al., 2013). Cell wall associated genes and potential peptidases were derived from the Black Yeast Genome Database (Moreno et al., 2017).

### Virulence in *Galleria mellonella*

Conidia cells were harvested from MEA-grown cultures incubated for 7 days at 33°C. Conidia were counted in a Bürker-Turk counting chamber and a standardized conidial suspension was made. This suspension consisted of 10^7^–10^4^ conidia per larvae. To verify the number of colony forming units, 40 μL of 10^3^ and 10^2^ inoculums were placed on SGA plates with gentamycin. Plates were incubated for 14 days at 37°C and colonies were counted. To inject the larvae, 40 μL of the conidia suspension was injected into the haemocoel of *G. mellonella* larvae via the last left proleg with an insulin micro-syringe. As a control in each experiment, one *G. mellonella* group was injected with PBS only. Each strain was tested in three independent experiments in separate weeks. Viability of the larvae was checked daily. Any cocoons formed, were disregarded and left out of the equations. Values were presented as the mean obtained from the three separate experiments and differences were analyzed with the Mann-Whitney *U*-test; statistical significance was set at *P* < 0.05 using GraphPad Prism 5 (GraphPad, La Jolla, CA, USA) and SPSS 19.0 (IBM, USA) softwares.

## Results

### Habitats of *E. dermatitidis* and *E. spinifera*

*Exophiala dermatitidis* is a relatively widespread fungus in the environment, although mostly occurring in low abundance. An extensive ecological study was performed by Sudhadham et al. (2008), who searched for the species in numerous natural and domestic environments and detected the species in fruit samples, feces of frugivorous birds, and natural hot springs, but particularly in steam baths and on creosoted railway sleepers. Other studies (Dogen et al., 2013a; Gümral et al., 2015; Zupancic et al., 2016) confirmed the prevalence in bathing facilities, on railway sleepers, and in dishwashers. Common fungal habitats as plant debris and soil were consistently negative or nearly so. Thus, the fungus seems selected by either toxic, or by hot, moist and nutritionally poor environments.

**Figure 1:**
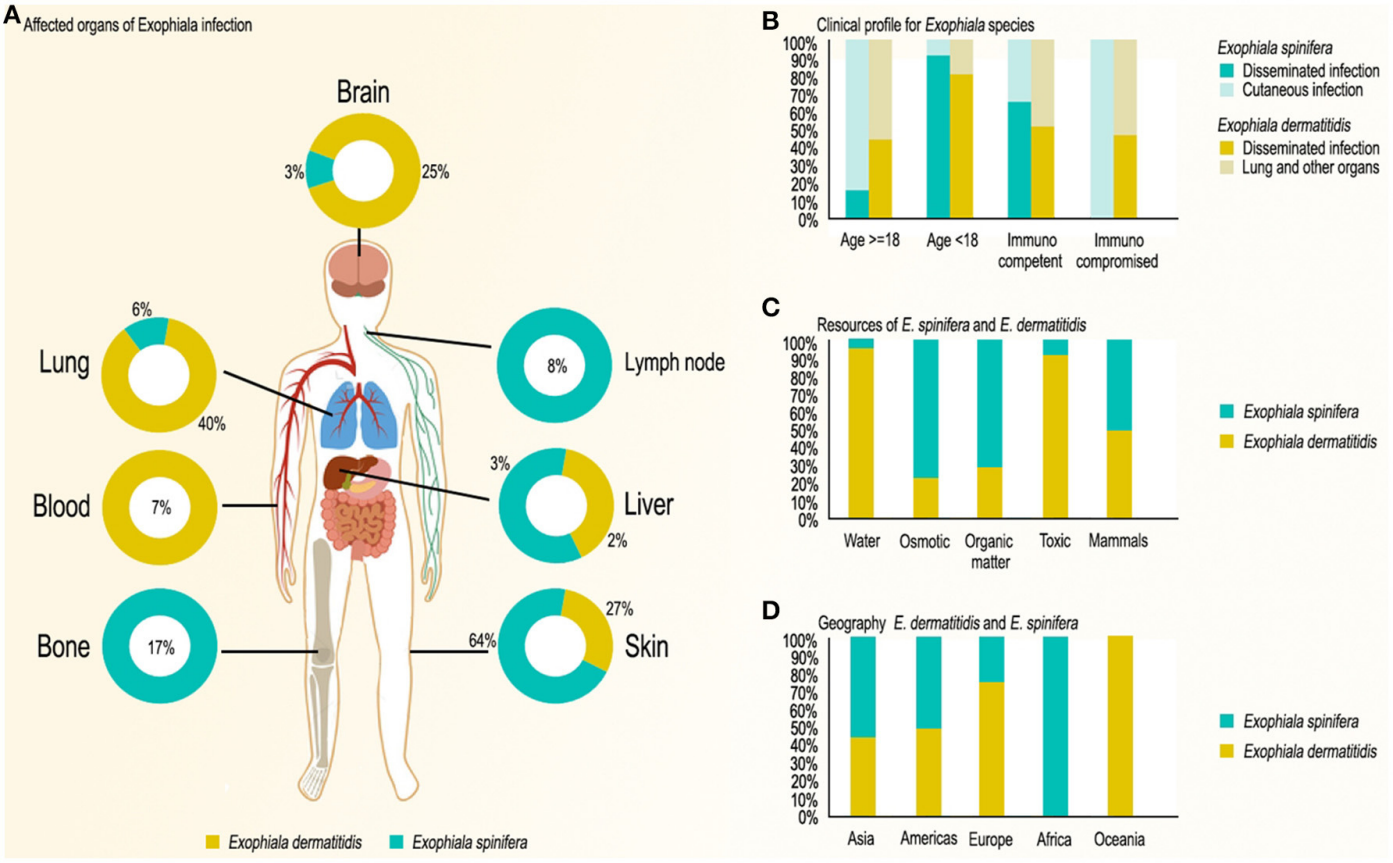
Infection and distribution characteristics of *E. spinifera* and *E. dermatitidis* based on published case reports. **(A)** Organs affected; **(B)** Main clinical profiles of *Exophiala* species; **(C)** Environmental sources of *Exophiala* species; **(D)** Geography of *Exophiala* species.

*Exophiala spinifera* is a rare fungus in the environment. We analyzed almost all strains globally available in the literature and the research database available at Westerdijk Institute (Table 1). Strains originated from plant materials with high sugar content, such as pineapple, maize, sugarcane, rotten cactus, and apple juice. The species was particularly prevalent on decomposing scales of babassu coconuts (Nascimento et al., 2017), which are rich in lipids, terpenes and aromatic hydrocarbons (Figure 1). Creosoted wood, bathing facilities and dishwashers, but also soil and plant debris were consistently negative. Thus, the fungus seems selected by somewhat osmotic environments; its prevalence in the coconut habitat requires further study.

In the human patient, *E. dermatitidis* occurs on (sub) cutaneous locations causing otitis externa, keratitis, and onychomycosis (Matsumoto et al., 1993; De Hoog et al., 2000a), but is more frequently found systemically, regularly colonizing the lungs of patients with cystic fibrosis (CF) (Horre et al., 2004; Chotirmall and McElvaney, 2014) and the intestinal tract of debilitated patients (Matos et al., 2002; de Hoog et al., 2005). Until January 2017, 77 cases (Table 2) have been reported in the English literature; 39 cases (Table 2) are single systemic infections (excluding pseudoepidemics). Life-threatening systemic infections occur in patients underlying disorders but also in otherwise healthy individuals (Alabaz et al., 2009) (51%, 39/77) particularly in those of Asian descent (Sudhadham et al., 2008) (62%, 24/39). Among the 39 systemic infections, 14 cases showed neurotropism while none was osteotropic; 34 of the cases concerned immunocompetent patiens. Two case series concerned injection of contaminated fluids into the bloodstream of patients, where neurotropism was frequently observed (Matsumoto et al., 1993). A case a fatality rate of over 80% was noted in systemic infections (Patel et al., 2013; Table 2).

**Table 2:** Overview of published cases due to *E. spinifera* and *E. dermatitidis*.

**First author/year**	**Age/Sex**	**Country**	**Initial site**	**Cutaneous**	**Extra-cutaneous**	**Immuno-suppression**	**Treatment**	**Outcome**	**Disease type**
***E. SPINIFERA***									
Rajam 1958	7/M	India	Face	Disseminated	Bone	No	Penicillin, streptomycin, nystatin, isoniazid	Death	CBM
Nielsen 1968	72/F	USA	Face	Unique	No	No	Surgery	CR	PHM
Padhye 1983	6/M	El Salvador	Face	Disseminated	Lung	No	AMB, KTC, 5-FC, ITC	PR	PHM
Padhye1984	60/M	USA	Arm	Regional	No	Yes	KTC, 5-FC	CR	PHM
Lacaz 1984	5/F	Brazil	Cutaneous	Unknown	Brain	Unknown	AMB	Unknown	PHM
Kotylo 1989	62/F	USA	Finger	Unique	No	Yes	Surgery, ITC	CR	PHM
Barba-Gomez 1992	49/M	Mexico	Finger	Unique	No	No	ITC, liquid nitrogen	CR	CBM
Mirza 1993	13/M	Pakistan	Unknown	Disseminated	No	No	AMB, 5-FC	PR	PHM
Padhye 1996	62/M	USA	Finger	Regional	No	Yes	5-FC, ITC, surgery	PR, relapse	CBM
Campos-Takaki 1994	12/M	Brazil	Face	Disseminated	Bone, LAD	No	AMB	Death	PHM
Oba 2000	66/F	Japan	Arm	Unique	No	No	ITC, heat therapy	PR	PHM
Rajendran 2003	12/F	India	Face	Disseminated	LAD	No	ITC	PR	PHM
Negroni 2004	32/F	Argentina	Face	Few, distant	Bone, LAD	Yes	ITC, AMB, PZC, surgery	CR	PHM
Dutriaux 2005	59/F	France1	Leg	Few, regional	No	Yes	VRC	PR	PHM
Takahara 2005	85/F	Japan	Arm	Regional	No	Yes	ITC, minocycline	CR	PHM
Develoux 2006	58/M	Senegal	Leg	Regional	No	No	TBF	Failure	CBM
Tomson 2006	78/M	Pakistan	Arm	Unique	No	No	ITC	Stability	CBM
Singal 2008	10/M	India	Leg	Disseminated	No	No	ITC, FCZ, TBF, cryo	Failure	PHM
Baubion 2008	73/M	France	Arm and leg	Multiple, Regional	No	Yes	ITC	Death	PHM
Chandler 2008	8/M	India	Leg	Disseminated	LAD	No	ITC, TBF	Failure	PHM
Harris 2009	49/M	USA	Leg	Few, regional	No	Yes	ITC	CR	PHM
Lin 2010	67/F	China	Scalp	Unknown	Unknown	Unknown	ITC	CR	PHM
Radhakrishnan 2010	20/F	India	Neck	Disseminated	Hepatic	No	KTC	Death	PHM
Li 2011	22/F	China	Face	Disseminated	Bone	No	Unknown	Death	PHM
Li 2011	9/M	China	Face	Disseminated	Bone	No	5-FC	Death	PHM
Badali 2012	55/M	India	Arm	Few, distant	No	No	Surgery	CR	PHM
Lin 2012	27/F	China	Leg	Multiple, regional	No	Yes	FLC	Death	PHM
Singh 2012	26/M	India	Face	Multiple, regional	No	No	ITC	PR	PHM
Daboit 2012	80/M	Brazil	Hand	Unique	No	No	ITC	CR	PHM
Badali 2012	55/M	India	Arm	Few, distant	No	No	Surgery	CR	PHM
Lanternier 2015	26/F	Iran	Face	Disseminated	Lungs	CARD9	FCZ, ITC, VRC	PR, relapse	PHM
Wang 2015	14/F	China	Trunk	Few, distant	No	No	ITC, ITC, TBF	CR	PHM
Wang 2015	23/F	China	Finger	Few, regional	No	No	5-FC	Lost to follow-up	PHM
Bohelay 2016	76/M	France	Finger	Unique	No	Yes	Surgery, ITC	Relapse, CR	PHM
Srinivas 2016	12/M	India	Foot	Disseminated	Bone and brain	No	ITC, VRC	CR	CBM
Wendy 2016	45/M	Brazil	Purulent subcutaneous cyst	Regional	No	Yes	ITC	CR	PHM
***E. DERMATITIDIS:***									
Hiruma 1993	24/M	Japan	Brain abscess	Disseminated	Brain	None	MCZ, 5-FC, AMPH-B, KTC	Death	PHM
Lye 1993	39/M	Singapore	Peritonitis	Disseminated	Peritonitis	Peritoneal dialysis	Catheter removal, FLC	NM	Peritonitis
Blaschke-Hellmessen 1994	3/M	Germany	Fungemia	Disseminated	blood	AML	Catheter removal, AMPB, 5-FC	Death	Acute leukemia
Ajanee 1996	70/M	Singapore	Brain abscess	Disseminated	Brain	None	AMB, Op	Death	PHM
Woollons 1996	58/F	UK	Phaeohyphomycosis	Regional	No	RA, steroid	ITC, Op	Cure	Lung cancer
Nachman 1996	3/M	USA	Fungemia	Disseminated	Brain	HIV	Catheter removal, AMB, ITC	Death	PHM
Chang 2000	28/M	Korea	Meningitis, brain abscess	Disseminated	Brain	None	AMB, Op	Death	PHM
Vlassopoulos 2001	53/F	Greece	Peritonitis	Disseminated	Peritonitis	Peritoneal dialysis	Catheter removal, FLC	Cure	Peritonitis
Diemert 2001	29/F	Canada	Pneumonia	Regional	NO	Cystic fibrosis	AMB, ITC, VRC	Cure	Cystic fibrosis
Liou 2002	62/M	Taiwan	Lymphadinitis	Disseminated	Unknown	AML	AMB, ITC	Relapse	Lymphadinitis
Myoken 2003	39/F	Japan	Invasive stomatitis	Disseminated	Peritonitis	AML	ITC, AMB	Cure	Stomatitis
Greig 2003	55/F	UK	Peritonitis	Disseminated	Peritonitis	Peritoneal dialysis	Catheter removal, AMB	Cure	Peritonitis
Tseng 2005	58/F	Taiwan	Fungemia	Disseminated	Blood	Lung cancer	Catheter removal, AMB	Cure	Catheter-related fungaemia
Mukaino 2006	54/F	Japan	Pneumonia	Regional	No	Bronchiectasis	MCZ, nebulized AMB	Death	Pulmonary disorder
Taj-Aldeen 2006	54/F	Netherlands	Pneumonia	Regional	No	DM, systemic cancer	FLC, ITC, AMB	Cure	Pneumonia
Ozawa 2007	81/F	Japan	Pneumonia	Regional	No	None	FLC, ITC	Cure	Pneumonia
Alabaz 2009	8/M	Turkey	Systemic phaeohyphomycosis	Disseminated	Unknown	None	AMB, VRC	Death	PHM
Chang 2009	3/M	China	Brain abscess, meningitis	Disseminated	Brain	None	AMB, FLC, ITC	Death	PHM
Hong 2009	11/F	Korea	Liver chirosis	Disseminated	Liver	None	VRC, liver transplant	Death	Liver cirrhosis
Oztas 2009	24/F	Turkey	Systemic phaeohyphomycosis	Disseminated	Unknown	None	AMB, VRC	Cure	PHM
Griffard 2010	16/F	USA	Pneumonia	Regional	No	Cystic fibrosis	ITC, VRC	Relapse	Pneumonia
Bulloch 2011	86/F	USA	Lung nodule	Regional	No	Dementia	VRC	Cure	Lung nodule
Russo 2010	17/M	Argentina	Phaeohyphomycosis	Regional	No	None	ITC, Op	Cure	PHM
Suzuki 2012	65/M	Japan	Lung nodule	Regional	No	Multiple myeloma	VRC, Op	Cure	Lung nodule
Li 2010	19/F	China	Meningitis	Disseminated	Brain	None	NM	Death	Meningitis
Li 2010	30/F	China	Meningitis	Disseminated	Brain	None	NM	Death	Meningitis
Li 2010	3/M	China	Meningitis	Disseminated	Brain	None	AMB, 5-FC	Death	Meningitis
Alabaz 2009	8/M	China	hepatic lesions	Disseminated	Lymph node	None	AMB, VRC	Death	Lymph node
CDC 2002	77/F	USA	Meningitis	Disseminated	Brain	Contaminated injectable steroids	AMB, VRC, 5-FC	Death	Meningitis
CDC 2002	61/F	USA	Meningitis	Disseminated	Brain	Contaminated injectable steroids	VRC	Cure	Meningitis
CDC 2002	71/F	USA	Meningitis	Disseminated	Brain	Contaminated injectable steroids	NM	NM	Meningitis
CDC 2002	65/F	USA	Meningitis	Disseminated	Brain	Contaminated injectable steroids	NM	NM	Meningitis
CDC 2002	52/F	USA	Meningitis	Disseminated	Brain	Contaminated injectable steroids	NM	NM	Meningitis
Greig 2003	55/M	USA	Peritonitis	Disseminated	Yes	CAPD	AMB, ITC	Cure	Peritonitis
Liou 2002	62/F	China	Lymph-adenitis	Disseminated	Yes	AML	AMB, ITC, FLC	Cure	Lymphadenitis
Kerkmann 1999	19/F	Germany	Chronic otitis	Disseminated	Yes	NM	NYS	Cure	Chronic otitis
Kabel 1994	5/M	Netherlands	Intravascular	Disseminated	Yes	No	ITC	Cure	AML
Kusenbach 1992	6/F	Germany	Pneumonia	Regional	Yes	No	ITC	Cure	Cystic fibrosis
Kenney 1992	21/F	USA	Systemic	Disseminated	Yes	No	AMB, FLC, KTC	Cure	Chronic granulomatous disease
Ventin 1987	63/M	Germany	Systemic	Disseminated	Yes	Intravenous drug abuse	AMB	Death	Valvular aortal prosthesis
Patel 2013	50/F	India	Invasive	Disseminated	No	NO	AMB, FLC, VRC, ITC	Cure	Native-valve endocarditis
Woollons 1996	58/M	UK	Subcutaneous	Regional	No	Steroid injection	ITC, skin grafting, surgery	Cure	Painful nodules on right hand
Crosby 1989	60/M	USA	Subcutaneous	Regional	No	NO	Op	Cure	Subcutaneous
Scott 1986	74/M	Australia	Subcutaneous	Regional	No	Burn wood	Op	Cure	PHM
Patel 2006	52/M	USA	Superficial	Regional	No	Keratomilensis	NATA, ITC, FLC	Cure	Keratitis
Benaoudia 1999	31/M	France	Superficial	Regional	No	Keratoplasty	Steroids, ITC	Cure	Keratitis
Pospisil 1990	51/F	Czechia	Superficial	Regional	No	NM	BFC	Cure	Melanonychia
Pospisil 1990	35/M	Czechia	Superficial	Regional	No	RecklingHausen	AMB, iodine, vitamins	NM	Keratitis
Matsumoto 1993	42/M	Japan	Nail	Regional	No	No	ITC	Cure	Toe nail
Matsumoto 1992	51/F	Japan	PHM	Regional	No	Diabetes	BFC	Cure	Toe nail
Sood 2014	21/M	India	Cerebral	Disseminated	Brain	NM	Op, AMB, VRC	Cure	Ear abscess
Ajanee 1996	70/M	Pakistan	Invasive	Disseminated	Brain	None	AMB, Op	Death	Brain abscess
Simpson 1995	53/F	UK	Peritoneal dialysis	Disseminated	Yes	Peritoneal dialysis	Catheter removal, FLC	Cure	Peritonitis
Haase 1990	54/F	Germany	Pneumonia	Regional	No	None	MCZ, nebulized AMB	Cure	Pneumonia
Crosby 1989	60/M	USA	Endophthalmitis	Regional	No	None	ITC, Op	Cure	Endophthalmitis
Hu 2014	8/M	China	Lung, CNS	Disseminated	No	None	5-FC, AMB, VRC	Cure	Pneumonia
Lanternier 2014	6/F	France	Brain, liver	Disseminated	Brain	CARD9-	NM	Relapse	NM
Chen 2016	78/M	China	Forearm	Regional	No	None	ITC	Cure	PHM
Watanabe 1961	41/M	Japan	Cheek	Regional	No	None	NM	Cure	PHM
Shimazono 1963	30/F	Japan	Brain	Disseminated	Brain	None	NM	Death	PHM
Sugawara 1964	15/M	Japan	Cheek	Disseminated	liver, gall bladder	Trauma	NM	Death	PHM
Tsai 2005	19/F	China	Cholelithiasis	Disseminated	Brain	Cholelithiasis	NM	Death	PHM
Harada 1989	15/M	Japan	Cutaneous	Disseminated	Lung	Cutaneous	NM	Death	PHM
Hohl 1983	65/M	USA	Subcutaneous	Regional	No	None	NM	Cure	PHM
Levenson 1984	29/M	USA	Cornea	Regional	No	Diabetes	NM	Cure	PHM
Crosby 1989	55/F	USA	Subcutaneous	Regional	No	Angina pectoris	NM	Cure	PHM
Barenfanger 1989	79/F	USA	Lung	Regional	No	Polymyositis	NM	Cure	PHM
Sharkey 1990	58/M	USA	Subcutaneous	Regional	No	Bronchiectasis	NM	Cure	PHM
Sharkey 1990	77/M	USA	Skin	Regional	No	Vasculitis	NM	Cure	PHM
Margo 1990	75/F	USA	Eye	Regional	No	Rheumatoid	NM	Cure	PHM
Crosby 1989	55/F	USA	Polymyositis	Disseminated	Blood	Polymyositis	NM	Death	PHM
Matsumoto 1990	34/M	Japan	Subcutaneous	Regional	No	Leukemia	NM	Cure	PHM
Myoken 2003	39/F	Japan	Gingiva	Regional	No	Leukemia	Op	Cure	PHM
Matsumoto 1992	51/F	Japan	Toe nail	Regional	No	None	BFC	Cure	PHM
Griffard 2010	16/F	USA	Acute respiratory exacerbations	Disseminated	Yes	Cystic fibrosis	VRC	Relapse	PHM
Diemert 2001	29/F	Canada	Lung	Disseminated	Yes	Cystic fibrosis	AMB, VRC	Cure	Invasive pulmonary
Kenney 1992	21/F	India	Cerebral	Disseminated	Brain	None	AMB, Op	Cure	Systemic infection

*Seventy seven E. dermatitidis cases and 36 E. spinifera cases have been reported in the English literature; main clinical characteristics are included*.

In contrast, *E. spinifera* is rarely involved in infections limited to the skin. Some cases (Rajam et al., 1958; Barba-Gomez et al., 1992; Padhye et al., 1996; Develoux et al., 2006; Tomson et al., 2006; Srinivas et al., 2016) were reported as chromoblastomycosis, although typical muriform cells were mostly lacking. Fatal systemic infections are known; 36 cases have been recorded in English and Chinese literature (Rajam et al., 1958; Nishimura and Miyaji, 1983; Padhye et al., 1983, 1984; Mirza et al., 1993; Campos-Takaki and Jardim, 1994; de Hoog et al., 1999; Rajendran et al., 2003; Negroni et al., 2004; Dutriaux et al., 2005; Baubion et al., 2008; Singal et al., 2008; Fothergill et al., 2009; Harris et al., 2009; Radhakrishnan et al., 2010; Li et al., 2011; Badali et al., 2012; Daboit et al., 2012; Lin et al., 2012; Bohelay et al., 2016; Silva et al., 2017; Table 2). Among the 12 systemic infections, all cases concerned immunocompetent patients. Osteotropism was mentioned in 5 cases, neurotropism in one case. An Asian predilection was notable (83%, 10/12). The fungus has not been encountered in CF lungs.

### Geography

*Exophiala dermatitidis* is known from natural habitats from tropical regions and has been reported in Brazil (Reiss and Mok, 1979), Nigeria (Muotoe-Okafor and Gugnani, 1993), and Thailand (Sudhadham et al., 2008; Zeng and De Hoog, 2008). In the domestic, man-made environment and in patients the fungus has a global distribution (Sudhadham et al., 2008; Zeng and De Hoog, 2008). Numerous isolates have been recorded from e.g., the following countries: Germany (Horre et al., 2004), Italy, Japan (Hiruma et al., 1993), Slovenia (Zalar et al., 2011), Sweden (Kondori et al., 2011), The Netherlands (de Hoog et al., 2005), Turkey (Dogen et al., 2013b), and USA (Zeng et al., 2007), covering several continents. *E. spinifera* was found to have a similar global distribution judging from literature and 48 available strains, with a slight bias to (sub) tropical regions: Argentina, Brazil, China, Colombia, Germany, India, Italy, Mexico, Papua New Guinea, Senegal, Thailand, and USA (Figure 1). Most of the isolates concerned patient materials.

### Diversity

*Exophiala dermatitidis* is known to have three main genotypes in ITS, viz. A, B, and C (Rath et al., 1997; Sudhadham et al., 2010), of which genotype A has been subdivided further (Rath et al., 1997). Single-gene trees of *TEF1* and *TUB* showed that there is random variation among partitions. This indicates that the published ITS haplotypes represent different variants of a single species. Haplotype diversity (Rozas and Rozas, 1995) for *E. dermatitidis* was as follows, *TEF1*: *h* = 10, Hd = 0.4825; *TUB*: *h* = 12, Hd = 0.6626, ITS: *h* = 10, Hd = 0.7077. The same parameters for *E. spinifera* were: *TEF1*: *h* = 10, Hd = 0.4541, *TUB*: *h* = 11, Hd = 0.8415, ITS: *h* = 12, Hd = 0.7606. No consistent difference was found in the multilocus tree between clinical and environmental strains, indicating that isolates from patients are likely to have an environmental origin. The nearest neighbor is *Exophiala phaeomuriformis*, which differs phenotypically by having a lower maximum growth temperature (Matos et al., 2003).

**Figure 2:**
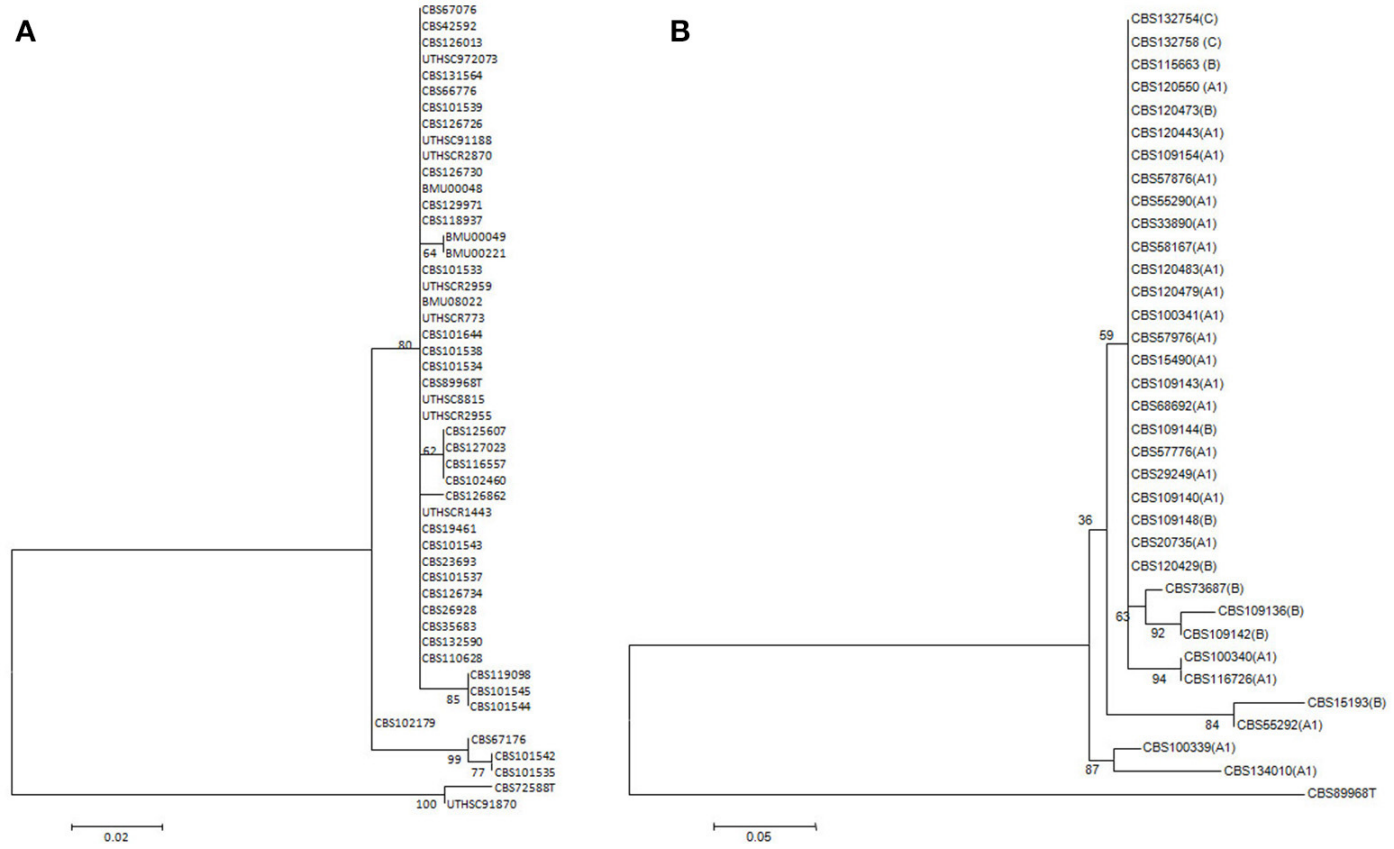
Dendrograms based on sequences of *TEF1* region of rDNA gene of *E. spinifera* and *E. dermatitidis*. Dendrograms are constructed using Maximum likelihood method with correction of Kimura 2 parameter (K2P) in the Bionumerics package. Bootstrap support calculated using 1,000 replicates. *Exophiala oligosperma* CBS 725.88 and UTHSC 91-870 were used as outgroup in the *E. spinifera* tree, *E. spinifera* CBS 899.68 in the *E. dermatitidis* tree. **(A)** *E. spinifera*. **(B)** *E. dermatitidi*s. Scale bars represent the estimated number of base substitutions per site.

The 48 strains of *E. spinifera*, when sequenced for ITS, *TUB* and *TEF1* did not show bootstrap-supported subclusters, and clustering was not concordant between genes. No consistent difference was found between clinical and environmental strains, which may indicate that the isolates from patients are likely to have an environmental origin (Figure 2). The nearest neighbor is *Exophiala exophialae*, represented by three strains differing in concordant ITS and *TEF1* sequences.

### Pathogenic traits

Results are presented in Table 3. Both species produce extracellular slime around 5–7 day-old budding cells at 24°C. In *E. spinifera* this occurs in the form of capsules of 5–6 μm width, of equal dimensions in environmental and clinical strains. The EPS produced under similar conditions in *E. dermatitidis* was diffused. Comparative genomic analyses using *E. spinifera, E. dermatitidis*, and *C. neoformans*, a model organism for capsule studies, revealed few differences between the black yeasts. Among the 62 genes correlated with the production and regulation of the polysaccharide capsule in *C. neoformans*, 45 and 42 orthologous genes were found in *E. spinifera* and *E. dermatitidis*, respectively (Table 4). Contrary to *E. spinifera*, where homologs of the alpha glucan synthase gene (AGS1) was found, in *E. dermatitidis* this homolog was absent, which might lead to slower growth, sensitivity to temperature and lack of structured capsule polysaccharide on budding cell surfaces (Reese et al., 2007). In addition, homologs corresponding to WSP1, a protein with multiple functions including production of the polysaccharide capsule and secretion of the enzyme urease (Shen et al., 2011), were identified in *E. spinifera* but seemed to be lost in the studied strain of *E. dermatitidis*.

**Table 3:** Physiological and other phenotypic test results of 20 selected strains of each species.

**Accession number**	**PST (mM)**	**5% NaCl**	**10% NaCl**	**5% MgCl_2_**	**10% MgCl_2_**	**Cycloheximide**	**Lipolysis**	**Proteolysis**	**Hemolysis**	**Urease**	**20% Sucrose**	**40% Sucrose**	**60% Sucrose**	**Cell shape 24°C**	**Cell shape 37°C**	**Capsule/EPS 24°C**	**Capsule/EPS 37°C**
***E. SPINIFERA***																	
CBS 101533	9	+	−	+	+	++	+	+	−	++	++	+	−	Meristematic	Meristematic	C	−
CBS 101539	9	+	−	+	+	++	+	+	+	w	++	+	−	Meristematic	Yeast	C	−
CBS 116557	9	+	−	+	+	++	+	+	−	+	++	+	−	Hyphae	Yeast	C	−
CBS 425.92	12	+	−	+	+	++	+	+	−	w	++	+	−	Meristematic	Yeast	C	−
CBS 667.76	9	+	−	+	+	++	+	+	−	++	++	−	−	Meristematic	Meristematic/Hyphae	C	−
CBS 670.76	9	+	−	+	+	++	+	+	+	−	++	−	−	combination	Hyphae	C	−
CBS 126013	12	+	−	+	+	++	+	+	+	++	++	−	−	Meristematic	Yeast	C	−
CBS 127023	12	+	−	+	+	++	+	+	+	++	++	+	−	Meristematic	Yeast	C	−
CBS126726	9	+	−	+	+	++	+	+	+	w	++	−	−	Meristematic	Yeast	C	−
CBS 131564	12	+	−	+	+	++	+	+	+	++	++	−	−	Yeast	Yeast	C	−
CBS 101543	9	+	−	+	+	++	+	+	+	++	++	−	−	Yeast	Yeast	C	−
CBS 102179	9	+	−	+	+	++	+	+	−	++	++	+	−	Meristematic	Yeast	C	−
CBS 119098	9	+	−	+	+	++	+	+	−	++	++	+	−	Meristematic	Meristematic	C	−
BMU 00048	9	+	+	+	+	++	+	+	+	−	++	+	−	Meristematic	Meristematic	C	−
BMU 00049	12	+	−	+	+	++	+	+	−	++	++	+	−	Hyphae	Hyphae/Meristematic	C	−
CBS 125607	12	+	−	+	+	++	+	−	+	W	++	+	−	Hyphae/Meristematic	Meristematic	C	−
CBS 129971	9	+	−	+	+	++	+	+	−	−	++	+	−	Meristematic	Hyphae	C	−
CBS 269.28	6	+	−	+	+	++	+	+	−	++	++	+	−	Meristematic	Hyphae	C	−
CBS 899.68	9	+	−	+	+	++	+	+	−	−	++	+	−	Meristematic	Meristematic	C	−
CBS 194.61	12	+	−	+	+	++	+	+	+	++	++	+	−	Meristematic	Meristematic	C	−
***E. DERMATITIDIS***																	
CBS 134010	6	+	−	+	−	+	−	−	−	++	++	+	−	Meristematic	Yeast/Hyphae	E	E
CBS 120483	9	+	−	+	−	+	−	−	−	−	++	−	−	Meristematic	Yeast/Hyphae	E	E
CBS 552.90	6	+	−	+	−	+	−	−	−	−	++	+	−	Yeast	Yeast	−	−
CBS 525.76	6	+	−	+	−	+	−	+	−	W	++	+	−	Yeast	Yeast	E	E
CBS 292.49	6	+	−	+	−	+	−	+	−	−	++	+	−	Meristematic	Meristematic	E	E
CBS 120443	6	+	−	+	−	+	−	−	−	−	++	+	−	Meristematic	Yeast	E	E
CBS 120550	6	+	−	+	−	+	−	−	−	−	++	+	−	Yeast	Yeast	E	E
CBS 578.76	9	+	−	+	−	+	−	−	−	−	++	+	−	Yeast	Yeast/Hyphae	E	E
CBS 115663	9	+	−	+	−	+	−	−	−	−	++	+	−	Meristematic	Meristematic	E	E
CBS 207.35	9	+	−	+	−	+	−	−	−	−	++	+	−	Yeast	Yeast	E	E
CBS 686.92	6	+	−	+	−	+	−	−	−	−	++	+	−	Yeast	Yeast	E	E
CBS 120429	6	+	−	+	−	+	−	−	−	W	++	−	−	very slow growth		E	E
CBS 120473	6	+	−	+	−	+	−	−	−	++	++	+	−	Yeast	Yeast	E	E
CBS 120472	9	+	−	+	−	+	−	−	−	−	++	+	−	Meristematic	Hyphae	E	E
CBS 109144	9	+	−	+	−	+	−	−	−	W	++	+	−	Meristematic	Meristematic	E	E
CBS 109149	9	+	−	+	−	+	−	−	−	−	++	−	−	Yeast/Hyphae	Yeast/Hyphae	E	−
CBS 132754	9	+	−	+	−	+	−	−	−	−	++	−	−	Yeast	Yeast	E	−
CBS 123474	6	+	−	+	−	+	−	−	−	−	++	−	−	Meristematic	Meristematic	E	−
CBS 132758	3	+	−	+	−	+	−	−	−	−	++	+	−	Yeast	Yeast	E	−
CBS 109154	9	+	−	+	−	+	−	−	−	−	++	+	−	Yeast	Yeast	E	−

*EPS, extracellular polysaccharide; PST, Peroxide stress tolerance (maximum concentration tolerated). W or w, weak positive; +, positive; ++, strong positive; −, negative*.

**Table 4:** Polysaccharide capsule production related genes.

***C. neoformans*-Gene ID**	**Name**	**Capsule phenotype**	**References**	***E. spinifera*-Gene ID**	***E. dermatitidis*-Gene ID**
CNAG_00125	*CRG1*	Hyper	Wang et al., 2004	PV08_00096T0	HMPREF1120_07029T0
CNAG_00268	*ILV2*	Hypo	Kingsbury et al., 2004	PV08_11571T0	HMPREF1120_02016T0
CNAG_00375	*GCN5*	Hypo	ÒMeara et al., 2010a	PV08_07822T0	HMPREF1120_05017T0
CNAG_00396	*PKA1*	Hypo	D'Souza et al., 2001	N/A	N/A
CNAG_00440	*SSN801*	Hyper	Liu et al., 2008	PV08_01597T0	HMPREF1120_01045T0
CNAG_00570	*BCY1*	Hyper	D'Souza et al., 2001	PV08_10852T0	HMPREF1120_06501T0
CNAG_00600	*CAP60*	Hypo	Chang and Kwon-Chung, 1998; Moyrand and Janbon, 2004	N/A	N/A
CNAG_00697	*UGE1*	Hyper	Moyrand et al., 2007	PV08_02096T0	HMPREF1120_01443T0
CNAG_00721	*CAP59*	Hypo	Chang and Kwon-Chung, 1994; Moyrand and Janbon, 2004	N/A	N/A
CNAG_00746	*CAS35*	Hypo	Moyrand et al., 2004, 2007	N/A	N/A
CNAG_00769	*PBS2*	Hyper	Bahn et al., 2005	PV08_02653T0	HMPREF1120_02538T0
CNAG_01106	*VPH1*	Hypo	Erickson et al., 2001	PV08_05620T0	HMPREF1120_06087T0
CNAG_01172	*PBX1*	Hypo	Liu et al., 2007	N/A	N/A
CNAG_01371	*CRG2*	Hyper	Shen et al., 2008	PV08_07821T0	HMPREF1120_05016T0
CNAG_01523	*HOG1*	Hyper	Bahn et al., 2005	PV08_06527T0	HMPREF1120_05833T0
CNAG_01551	*GAT201*	Hypo	Liu et al., 2008	PV08_04845T0	HMPREF1120_00248T0
CNAG_01626	*ADA2*	Hypo	Haynes et al., 2011	PV08_00126T0	HMPREF1120_06980T0
CNAG_01654	*CAS34*	GXM defect	Moyrand et al., 2007	N/A	N/A
CNAG_01727	*SSA1*	Hyper	Zhang et al., 2006	PV08_02973T0	HMPREF1120_02626T0
CNAG_01845	*PKC1*	Hypo	Heung et al., 2005	PV08_09341T0	HMPREF1120_07353T0
CNAG_01890	*MET6*	Hypo	Pascon et al., 2004	PV08_04416T0	HMPREF1120_05363T0
CNAG_02029	*WSP1*	Hypo	Shen et al., 2011	PV08_03067T0	N/A
CNAG_02153	*TUP1*	Hyper	Lee et al., 2009	PV08_11209T0	HMPREF1120_04775T0
CNAG_02215	*HAP3*	Hypo	Jung et al., 2010	PV08_04195T0	N/A
CNAG_02236	*PPG1*	Hypo	Gerik et al., 2005	PV08_01602T0	HMPREF1120_01040T0
CNAG_02702	*GEF1*	Hypo	Zhu and Williamson, 2003	PV08_01769T0	HMPREF1120_01007T0
CNAG_02797	*CPL1*	Hypo	Liu et al., 2008	N/A	N/A
CNAG_02885	*CAP64*	Hypo	Chang et al., 1996; Moyrand and Janbon, 2004	N/A	N/A
CNAG_03120	*AGS1*	Hypo	Reese et al., 2007	PV08_03292T0	N/A
CNAG_03202	*CAC1*	Hypo	Alspaugh et al., 2002	PV08_06497T0	HMPREF1120_06227T0
CNAG_03322	*UXS1*	Xylosolation defect	Moyrand et al., 2002	N/A	N/A
CNAG_03438	*HXT1*	Hyper	Chikamori and Fukushima, 2005	N/A	N/A
CNAG_03582	*RIM20*	Hypo	ÒMeara et al., 2010b	PV08_05544T0	HMPREF1120_02658T0
CNAG_03670	*IRE1*	Hypo	Cheon et al., 2011	PV08_04820T0	HMPREF1120_00610T0
CNAG_03818	*SSK1*	Hyper	Bahn et al., 2007	PV08_04132T0	HMPREF1120_04973T0
CNAG_04162	*PKA2*	Hypo	D'Souza et al., 2001	PV08_06181T0	HMPREF1120_06255T0
CNAG_04312	*MAN1*	Hypo	Wills et al., 2001	PV08_06851T0	HMPREF1120_08308T0
CNAG_04505	*GPA1*	Hypo	Alspaugh et al., 1997	PV08_06528T0	HMPREF1120_05834T0
CNAG_04730	*GPR4*	Hypo	Xue et al., 2006	N/A	N/A
CNAG_04864	*CIR1*	Hypo	Jung et al., 2006	PV08_09234T0	HMPREF1120_00896T0
CNAG_04952	*CPS1*	Hypo	Chang et al., 2006	PV08_07536T0	HMPREF1120_08949T0
CNAG_04969	*UGD1*	Hypo	Griffith et al., 2004; Moyrand and Janbon, 2004	PV08_08061T0	HMPREF1120_03278T0
CNAG_05081	*PDE1*	Hyper	Hicks et al., 2005	N/A	N/A
CNAG_05139	*UGT1*	Hyper	Moyrand et al., 2007	PV08_01002T0	HMPREF1120_07559T0
CNAG_05218	*ACA1*	Hypo	Bahn et al., 2004	PV08_06686T0	HMPREF1120_08701T0
CNAG_05222	*NRG1*	Hypo	Cramer et al., 2006	N/A	N/A
CNAG_05431	*RIM101*	Hypo	ÒMeara et al., 2010b	PV08_06048T0	HMPREF1120_00699T0
CNAG_05562	*PBX2*	Hypo	Liu et al., 2007	N/A	N/A
CNAG_05563	*HOS2*	Hyper	Liu et al., 2008	N/A	N/A
CNAG_05581	*CHS3*	Hyper	Baker et al., 2007, Banks et al., 2005	PV08_01895T0	HMPREF1120_07721T0
CNAG_05703	*LRG1*	Hypo	Gerik et al., 2005	PV08_01033T0	HMPREF1120_07444T0
CNAG_05817	*GMT1*	Hypo	Cottrell et al., 2007	PV08_03821T0	HMPREF1120_04904T0
CNAG_06301	*SCH9*	Hyper	Wang et al., 2004	PV08_11389T0	HMPREF1120_01691T0
CNAG_06591	*SET302*	Hyper	Liu et al., 2008	N/A	N/A
CNAG_06808	*CPRa*	Hypo	Chang et al., 2003	N/A	N/A
CNAG_07408	*STE20*	Hypo	Wang et al., 2002	PV08_01113T0	HMPREF1120_05115T0
CNAG_07470	*PDE2*	Hyper	Hicks et al., 2005	PV08_05494T0	HMPREF1120_02921T0
CNAG_07554	*CAP10*	Hypo	Chang and Kwon-Chung, 1999; Moyrand and Janbon, 2004	PV08_06099T0	HMPREF1120_00655T0
CNAG_07636	*CSR2*	Hyper	Baker et al., 2007, Banks et al., 2005	PV08_01896T0	HMPREF1120_07720T0
CNAG_07680	*HAP5*	Hypo	Jung et al., 2010	PV08_04111T0	HMPREF1120_05506T0
CNAG_07718	*CIN1*	Hypo	Shen et al., 2010	PV08_01341T0	HMPREF1120_00287T0
CNAG_07937	*CAS1*	O-acetylation defect	Janbon et al., 2001	PV08_08786T0	N/A

**Figure 3:**
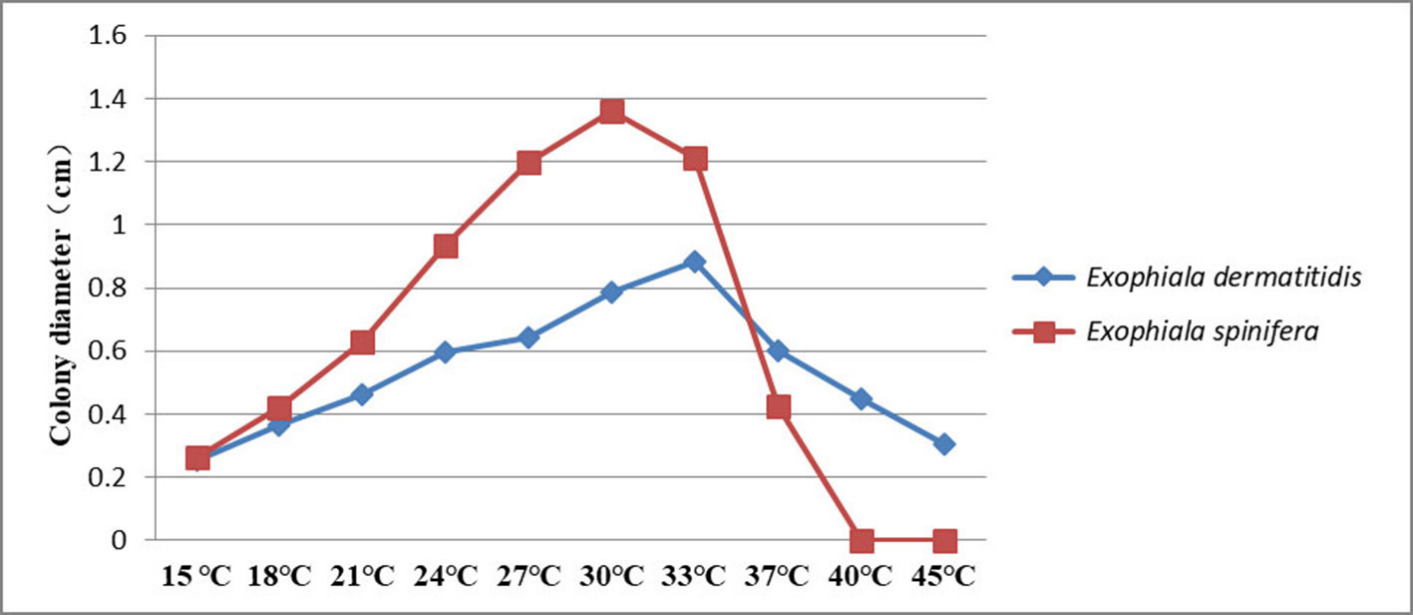
Thermotolerance of *E. spinifera* and *E. dermatitidis*. The averaged over 20 selected strains of each species are tested.

Both species had a broad range of thermotolerance (Figure 3). *E. dermatitidis* has an optimum at 33°C and still is able to grow at 45°C. *E. spinifera* has an optimum at 30°C, while its maximum growth temperature is 40°C. No growth is observed at 45°C; this temperature is fungistatic as about 50% of the strains showed regrowth when placed at 24°C.

**Figure 4:**
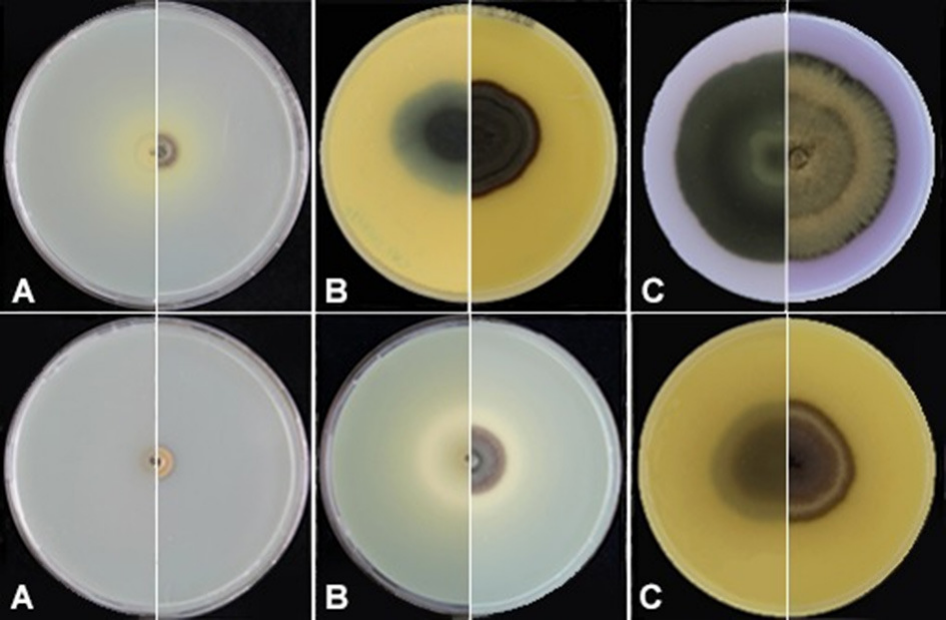
Proteolysis of *E. spinifera* strain CBS 125607 (upper panel) and *E. dermatitidis* strain CBS 120483 (lower panel). **(A)** Incubation for 1 week; **(B)** Incubation for 2.5 weeks; **(C)** Incubation for 1 month.

Hemolysis remained negative in all strains of *E. dermatitidis* and was positive in 50% of *E. spinifera* strains. *E. dermatitidis* showed slow acidification (yellow color change) and no proteolysis on Bromocresol purple-milk solids-glucose agar (BCP-MS-G). *E. spinifera* showed acidification within 1–2 weeks followed by a change to purple (alkaline) indicating casein decomposition (Figure 4). In general, growth velocity of *E. spinifera* was stimulated on protein medium. Nine strains of *E. spinifera* showed acidification of calcium carbonate medium, while only two *E. dermatitidis* were positive. Urease was positive in 25% of *E. dermatitidis* strains and in 80% of *E. spinifera* strains. The repertory of genes associated with nitrogen metabolism is composed by a single copy of the gene coding for urease (URE1) in both *E. dermatitidis* and *E. spinifera* (Table 5). In *E. dermatididis*, the homolog gene of URE1 shares 86% of BLASTP identity with that of the neurotropic black fungus *Rhinocladiella mackenziei*. The URE1 found in *E. spinifera* is highly conserved among other black yeasts members of the jeanselmei-clade, including *Exophiala oligosperma* (94% identity) and *Exophiala xenobiotica* (89% identity).

**Table 5:** Ureases and their associated genes.

Gene^*^	**Z518_04527-UreF**	**Z518_02869-UreD**	**Z518_06116-UreG**	**Z518_07397-Nickel/cobalt transporter, high-affinity**	**Z518_09873-URE1**
*E. dermatitidis*	HMPREF1120_02686T0	HMPREF1120_00803T0	HMPREF1120_08744T0	HMPREF1120_08266T0	HMPREF1120_06619T0
*E. spinifera*	PV08_05586T0	PV08_01642T0	PV08_07272T0	PV08_07662T0	PV08_10669T0

* *Reference genes extracted from R. mackenziei*.

**Figure 5:**
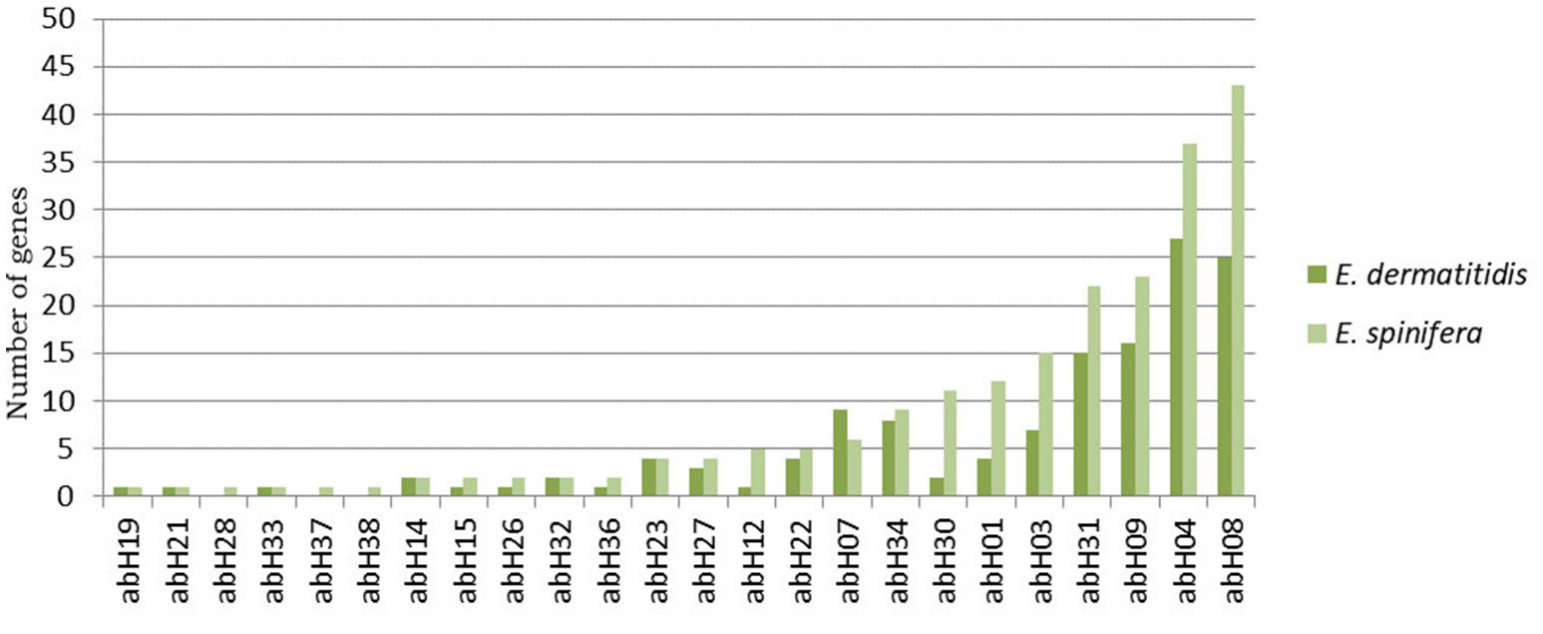
Lipase composition in *E. spinifera* and *E. dermatitidis*. Twenty-one lipase families are conserved among these fungi, while three families seem to be specific to *E. spinifera*.

Lipolysis was positive in all *E. spinifera* strains and negative in *E. dermatitidis* strains. Genome-wide analyses revealed an abundance of lipases in both *E. dermatididis* and *E. spinifera*. Overall, the species possess 135 and 212 putative lipases, respectively. Twenty-one lipase families are conserved among these fungi, while three families seem to be specific to *E. spinifera* (Figure 5). The most abundant superfamily in *E. spinifera* was annotated ascytosolic hydrolases (abH08), including epoxide hydrolases and haloalkane dehalogenases. In *E. dermatididis*, the most abundant superfamily corresponds to Moraxella lipase 2-like (abH04). The superfamily abH04 consists of three families of bacterial esterases and lipases.

Both species grow well on 5% NaCl or MgCl_2_, while *E. spinifera* tolerated 10% MgCl_2_. No osmotolerance (60% sucrose) was observed. Cycloheximide tolerance was noted in both species, growth of *E. spinifera* being better than that of *E. dermatitidis*. Highest degrees of tolerance, with growth on SGA with 0.2% cycloheximide at 37°C, was observed in *E. spinifera*, while *E. dermatitidis* was somewhat inhibited. Growth at ambient temperature was with hyphae and yeast cells with a small number of isodiametrically inflating (meristematic) cells with dark and thick walls resembling muriform cells, but at 37°C yeast cells were preponderant. Incubated in acidic medium at 24 and 37°C, no septate muriform cells were produced, but dark, thick-walled yeast-like cells were prevalent. Comparative analyses of the cell wall genes in *E. spinifera* against the previously described gene set in *E. dermatitidis* revealed important differences between the species. In contrast to *E. dermatididis, E. spinifera* possesses multiple copies of genes involved in α-glucan metabolism (Table 6). For instance, the GT5 and GH13 genes (PV08_03291, PV08_03292, and PV08_03826), which were reported to be reduced in *E. dermatitidis*, were found in *E. spinifera*. These genes are required for the 1, 3-α-glucan synthesis. Conversely, all seven previously described chitin synthase genes were observed in both species (Table 6).

**Table 6:** Cell wall modification pathway genes.

Genes^*^: the key or differential genes in the cell wall modification pathway	***E. dermatitidis***	***E. spinifera***
Chitin synthase		
CHS1 Class I, CHS2 class II	HMPREF1120_06816, HMPREF1120_07981	PV08_00820, PV08_10842
CHS3 Class III	HMPREF1120_06479	PV08_10744
CHS4 Class IV	HMPREF1120_07721	PV08_01895
CHS5 Class V, CHS7 Class VII	HMPREF1120_08776, HMPREF1120_08777	PV08_07002, PV08_07003
CHS6 Class VI	HMPREF1120_09115	PV08_07085
Chitin synthase like	HMPREF1120_01791	
UDP-N-acetylglucosamine 6-dehydrogenase	HMPREF1120_01790	PV08_06692
Regulation of chitin synthase activity, by analogy to *S. cerevisiae*		
SKT5 Activator of Chs3p during vegetative growth	HMPREF1120_07720	PV08_01896
Similarity with ScSkt5, activator of Chs3	HMPREF1120_06335	PV08_10210
Similarity with ScSkt5, activator of Chs3	HMPREF1120_05528	PV08_11174
BNI4 scaffold protein that tethers chitin synthase III (Chs3p) to the bud neck	HMPREF1120_05249	PV08_03080
ScCHS5 Similarity with ScChs5, component of exomer complex	HMPREF1120_05359	PV08_04423
ScCHS6 Similarity with ScChs6, component of exomer complex	HMPREF1120_01856	PV08_11343
Similarity with export control protein ScChs7	HMPREF1120_00837	PV08_09293
ScCHS7 Similarity with export control protein ScChs7	HMPREF1120_03003	PV08_09547
Chitin modification		
Cda1/2 chitin deacetylase	HMPREF1120_08023	PV08_03824
Chitin degradation		
ChiA GPI anchored class III chitinase	HMPREF1120_03399	PV08_09305
Class III chitinase	HMPREF1120_02334	PV08_02554
ChiB Class V chitinase	HMPREF1120_06669	PV08_07861
Class V chitinase	HMPREF1120_03714	
Chitinase	HMPREF1120_04557	PV08_03599
Chitinase	HMPREF1120_07241	
NagA Extracellular N-acetyl-beta-glucosaminidase	HMPREF1120_06035	PV08_05624
NagA	HMPREF1120_06285	PV08_06135
1,3-a-glucan synthesis and processing		
AgsB/A Catalytic subunits of the 1,3-a-glucan synthase complex (GT5 and GH13) - http://www.cazy.org/		PV08_03291, PV08_03292, PV08_03826
	HMPREF1120_08319	PV08_06861
Putative amylase; similarity with H. capsulatum Amy1	HMPREF1120_03460	PV08_02428
1,3-b-glucan synthesis and processing		
FksA Putative catalytic subunit 1,3-b-glucan synthase complex; ScFks1-like	HMPREF1120_03476	PV08_10508
ScSMI1 Putative regulatory component 1,3-b-glucan synthesis; ScKnr4-like	HMPREF1120_04893	PV08_03822
EngA Endo-1,3-b-glucanase (GH 81-family); ScEng1-like	HMPREF1120_09022	PV08_06894
Putative exo-1,3-b-glucanase family (GH 5); related to the ScExg1-family	HMPREF1120_04506	PV08_03500
	HMPREF1120_06180	PV08_06271
Putative exo-1,3-b-glucanase family (GH 55); related to Coniothyrium minitans exo-1,3-glucanase (Cmg1)	HMPREF1120_01556	PV08_03602, PV08_11776
	HMPREF1120_05230	
Bgl2-family of putative 1,3-b-transglucosylases (GH 17) proposed to be involved	HMPREF1120_00547	PV08_01318
	HMPREF1120_05209	PV08_00062
	HMPREF1120_06595	PV08_10359
	HMPREF1120_08449	PV08_01193
	HMPREF1120_04141	PV08_08282
	HMPREF1120_03066	PV08_03009
	HMPREF1120_08078	PV08_10096
Crh1-family of putative transglycosidases (GH 16);involved in crosslinking b-glucan	HMPREF1120_04931	PV08_03356
	HMPREF1120_00627	PV08_04702
	HMPREF1120_07927	PV08_07455
	HMPREF1120_02703	PV08_05520
Gas-family of putative 1,3-b-transglucosylases (GH 72) proposed to be involved in connecting the emerging 1,3-b-glucan chains to the existing b-glucan	HMPREF1120_07283	PV08_09503
	HMPREF1120_01763	PV08_11789
	HMPREF1120_03477	PV08_10507
GelG 1,3-b-glucanosyltransferase	HMPREF1120_01682	PV08_11613
SunA Sun family, involved in septation, possibly b-glucosidase activity;	HMPREF1120_01649	PV08_06721
SunB	HMPREF1120_06902	PV08_00042
Kre6 Putative transglycosidase required for 1,6-b-glucan biosynthesis	HMPREF1120_01614	PV08_06798
CelA Similarity with cellulose synthases of the GT 2 family. Putatively involved in 1,3-b-/1,4-b-glucan synthesis	HMPREF1120_04699	PV08_00315
Mlg1 Mixed-linked glucanases in C. carbonum, hydrolyze 1,3-b-/1,4-b-glucans	HMPREF1120_05299	
Mlg1	HMPREF1120_02373	PV08_09397
Mlg1	HMPREF1120_07765	PV08_01870
	HMPREF1120_09051	PV08_07166
Other cell wall biosynthesis proteins		
Endo-mannanase family (GH 76) with a putative role in GPI-CWP incorporation;	HMPREF1120_04431	PV08_03378
	HMPREF1120_03513	PV08_10453
	HMPREF1120_05522	PV08_11178

**Figure 6:**
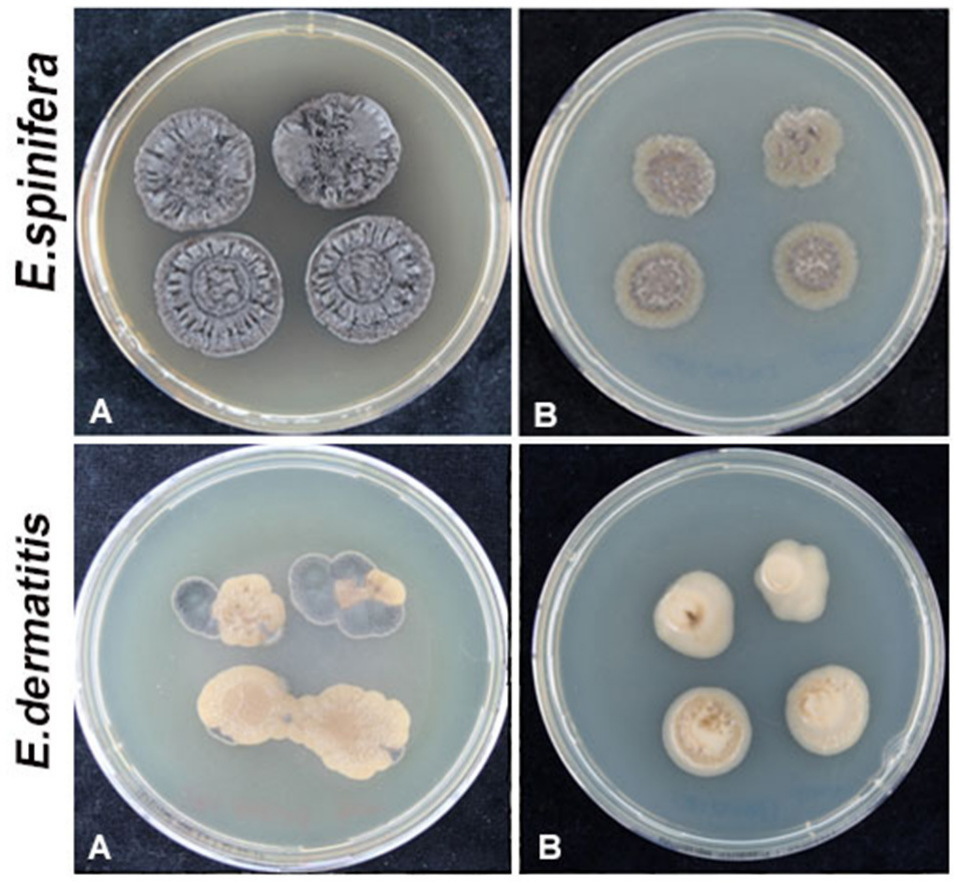
Peroxide tolerance for *E. spinifera* strain CBS 101542 and *E. dermatitidis* strain CBS 134010. Upper panel **(A,B)** melanized colonies of CBS 101542 subjected to CBS 101542 peroxide; Lower panel **(A,B)** colonies subjected to CBS 134010 peroxide showing loss of melanin.

**Figure 7:**
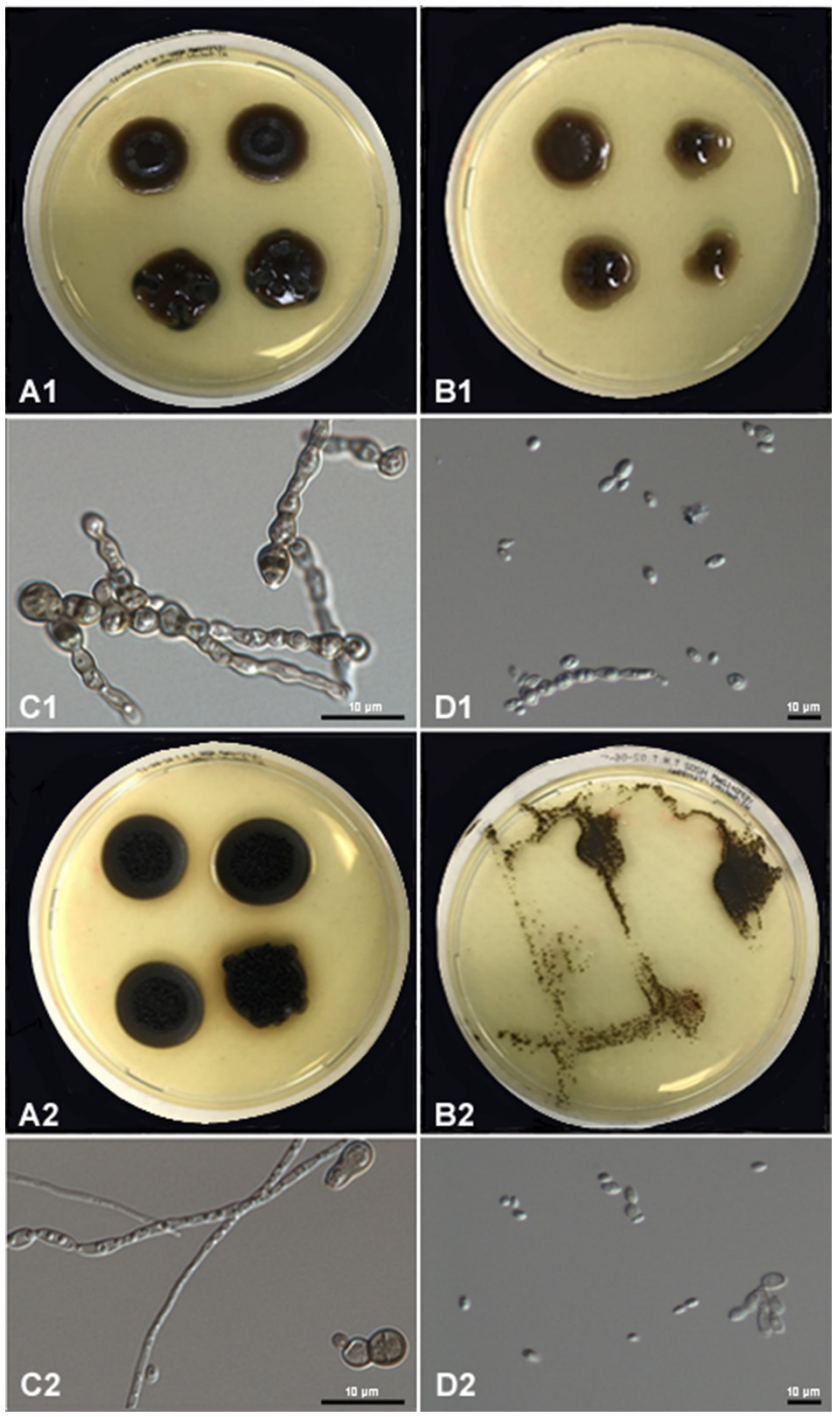
Peroxide tolerance of *E. spinifera* strain CBS 116557 (upper two panels) and *E. dermatitidis* strain CBS 115663 (lower two panels). **(A)** Show mycelial phase, **(B)** show yeast phase, **(C)** show muriform cell, **(D)** show yeast cell.

**Figure 8:**
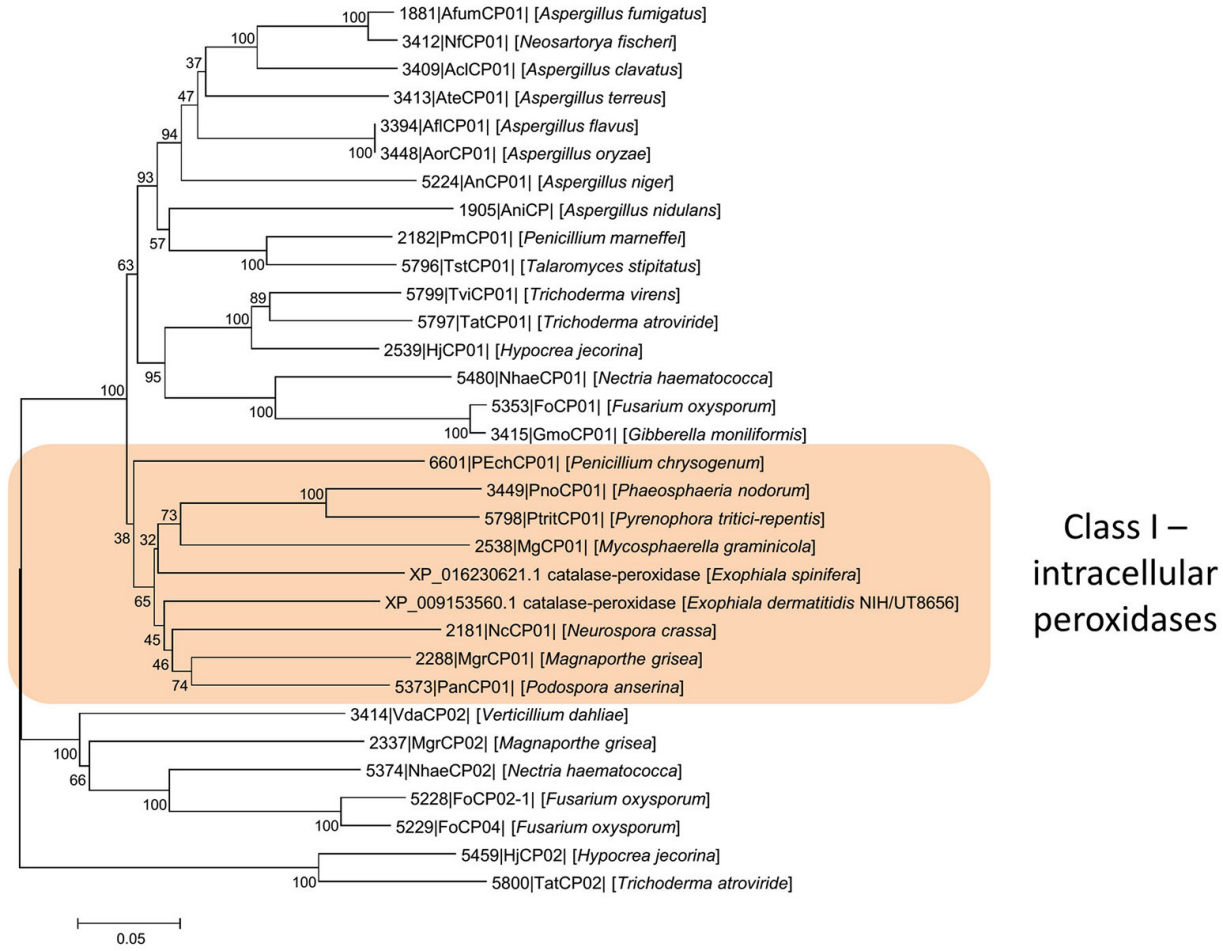
Phylogenetic analysis of intracellular peroxidases. The analysis indicates that they belong to Class I, which includes intracellular peroxidases involved in cellular protection against toxic peroxides. Scale bars represent the estimated number of base substitutions per site.

*Exophiala dermatitidis* tolerated 6–9 mM hydrogen peroxide, while the maximum concentration allowing growth of *E. spinifera* was 9–12 mM. With increasing H_2_O_2_-concentration, morphology of colonies changed from hyphae to yeast and cell walls tended to lose melanin (Figures 6, 7 and Tables 7, 8). In both species we predicted genes coding for the bifunctional catalase/peroxidase enzymes (PV08_11368 in *E. spinifera* and HMPREF1120_01299 in *E. dermatitidis*) that may reduce hydrogen peroxide. The genes carry the functional protein domain Haem peroxidase (IPR002016). Phylogenetic analyses indicated that it belongs to Class I, which includes intracellular peroxidases involved in cellular protection against toxic peroxides (Delort et al., 1989; Figure 8).

**Table 7:** The assimilation responses of 40 strains to different compounds.

**No**.	**CBS number**	**Hydrogen peroxide**	**Tween-80**	**Calcium carbonate**	**Urea**	**Sucrose**	**Sodium**	**Cycloheximide**	**Casein**
***E. DERMATITIDIS***									
1	CBS134010	+	−	−	+	+	+	+	−
2	CBS 120483	+	−	+	−	+	+	+	−
3	CBS 552,90	+	−	−	−	+	+	+	−
4	CBS207.35	+	−	−	−	+	+	+	−
5	CBS525.76	+	−	−	+	+	+	+	−
6	CBS292.49	+	−	−	−	+	+	+	−
7	CBS 120443	+	−	−	−	+	+	+	−
8	CBS 120550	+	−	−	−	+	+	+	−
9	CBS 578,76	+	−	−	−	+	+	+	−
10	CBS115663	+	−	−	−	+	+	+	−
11	CBS 686,92	+	−	−	−	+	+	+	−
12	CBS 120429	+	−	−	+	+	+	+	−
13	CBS 120473	+	−	+	+	+	+	+	−
14	CBS 120472	+	−	−	−	+	+	+	−
15	CBS 109144	+	−	−	+	+	+	+	−
16	CBS 109149	+	−	−	−	+	+	+	−
17	CBS 132754	+	−	−	−	+	+	+	−
18	CBS 123474	+	−	−	−	+	+	+	−
19	CBS 132758	+	−	−	−	+	+	+	−
20	CBS 109154	+	−	−	−	+	+	+	−
***E. SPINIFERA***									
1	CBS 101533	+	+	+	+	+	+	+	+
2	CBS 101539	+	+	+	+	+	+	+	+
3	CBS 116557	+	+	+	+	+	+	+	+
4	CBS 425.92	+	+	−	+	+	+	+	+
5	CBS 669.76	+	+	−	+	+	+	+	+
6	CBS 671.76	+	+	−	+	+	+	+	+
7	CBS 126013	+	+	+	−	+	+	+	+
8	CBS 127023	+	+	+	+	+	+	+	+
9	CBS126726	+	+	−	+	+	+	+	+
10	CBS 131564	+	+	−	+	+	+	+	+
11	CBS 101543	+	+	−	+	+	+	+	+
12	CBS 102179	+	+	+	+	+	+	+	+
13	CBS 119098	+	+	−	+	+	+	+	+
14	CBS 123468	+	+	−	+	+	+	+	+
15	CBS 123469	+	+	−	−	+	+	+	+
16	CBS 125607	+	+	+	+	+	+	+	+
17	CBS 129971	+	+	+	+	+	+	+	+
18	CBS 269.28	+	+	−	−	+	+	+	+
19	CBS 899.68	+	+	+	+	+	+	+	+
20	CBS 194.61	+	+	−	−	+	+	+	+

*+, positive; −, negative*.

**Table 8:** Responses of 20 strains of *E. spinifera* and 20 of *E. dermatitidis* upon peroxide challenge.

**No. of strains at max. concentration tolerated**	**3 mM**	**6 mM**	**9 mM**	**12 mM**
*E. spinifera*	0	1	10	9
*E. dermatitidis*	1	10	9	0
**Yeast conversion at max. concentration**		+		−
*E. spinifera*	11		9	
*E. dermatitidis*	16		4	
**Melanization at max. concentration**		+		−
*Exophiala spinifera*	15		5	
*Exophiala dermatitidis*	4		16	

*+, positive; −, negative*.

### *Galleria mellonella* virulence model

**Figure 9:**
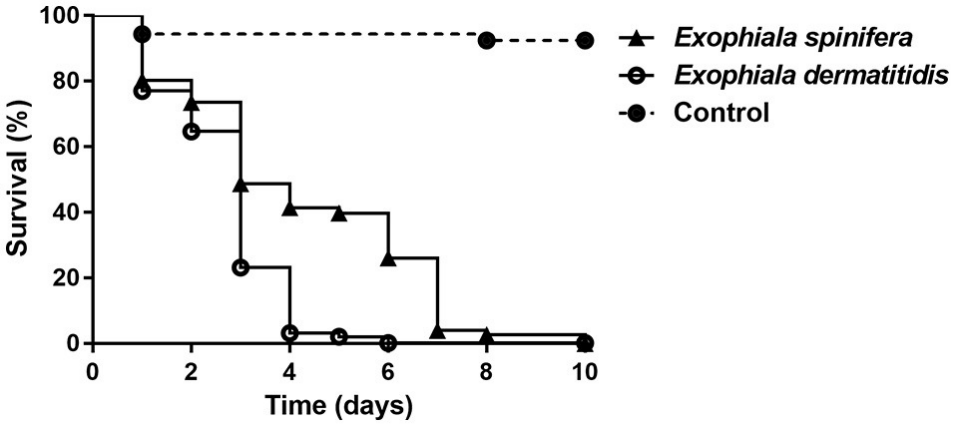
*Galleria mellonella* infection model of 10 strains for *E. spinifera* and 10 strains of *E. dermatitidis* tested at an inoculum density of 10^7^ cells. No significant differences were observed between clinical and environmental strains.

To determine if there was any difference in pathogenicity between *E. dermatitidis* and *E. spinifera*, we determined the LD50 in *Galleria mellonella* larvae at 37°C. No significant difference in the LD50 values between *E. dermatitidis* and *E. spinifera* was observed. However, we did notice a difference in death rates between the species. At an inoculum of 10^7^ Colony Forming Units per larvae, all larvae infected with *E. dermatitidis* died before day 6, while at that time point still 30% of *E. spinifera* infected larvae were alive. It took till day 10 before all *E. spinifera* infected larvae died. This difference in time-to-death between the two species was significant (Log-Rank, *p* = 0.001) (Figure 9). Within each species also a difference was noted between larvae infected with environmental isolates or with isolates obtained from clinical cases. The time-to-death in *G. mellonella* larvae infected with clinical isolates of *E. dermatitidis* was shorter than in *G. mellonella* larvae infected with environmental isolates of the same species (Log-Rank, *p* = 0.006). The same was true for *E. spinifera* (Log-Rank, *p* = 0.0326).

## Discussion

Melanized fungi of the order *Chaetothyriales* are frequently involved in human infection; the *Atlas of Clinical Fungi* lists 48 clinically relevant species. Nonetheless they are regarded as opportunists, as for only very few species a natural life cycle with an animal host has been suggested (Vicente et al., 2008). As opportunists, it is predicted that their invasive potential has to be explained from their environmental behavior. For example, agents of subcutaneous skin disease may be present on decaying thorns of prickly plants (Vicente et al., 2008), while hydrophilic yeast-like species can be carried by aerosols and are easily inhaled (Rath et al., 1997; Horre et al., 2004; Kondori et al., 2011). It is generally assumed that within these major categories of infection routes there is not much difference between species, the clinical course mainly being determined by portal of entrance and conditions of the host. In order to test this hypothesis, we compared two black yeast species with broad similarity in their growth form and infective ability, both being able to cause disseminated infections in immunocompetent humans. A simple overview of the literature learnt that the two species are clinically very different. *E. dermatitidis* is common as a pulmonary colonizer in patients with cystic fibrosis, where *E. spinifera* has never been observed.

The traumatic route may lead to subcutaneous infection. We found that both species tend to convert to yeast under hyperoxygen or temperature stress. Thus, mycetoma formation, as reported in *Exophiala jeanselmei* and exceptionally in *Exophiala oligosperma*, is less likely in *E. dermatitidis* and *E. spinifera*. Mycetoma grains are dense clumps of sterile hyphae which are not easily phagocytosed and provoke severe inflammation. Rather, the yeast conversion of *E. dermatitidis* and *E. spinifera* suggests potential dissemination in the bloodstream and is consistent with the clinical observation of disseminated infection.

Although both species are able to cause disseminated infections in otherwise apparently healthy hosts, *E. dermatitidis* regularly (36%) shows neurotropism, whereas this has never been observed in *E. spinifera*; in contrast, in the latter species some osteotropism (42%) has been mentioned (Rajam et al., 1958; Campos-Takaki and Jardim, 1994; Li et al., 2011; Srinivas et al., 2016).

The habitat choice of each fungus should explain observed types of opportunism on the human host (Vicente et al., 2008; Dogen et al., 2013b; Gumral et al., 2014). Our strain data show that the ecological differences between *E. spinifera* and *E. dermatitidis* are large. *E. dermatitidis* is found in habitats that are either toxic or poor in nutrients, suggesting evasion of microbial competition as a strategy of the fungus. In contrast, habitat choices of *E. spinifera* suggest some osmophily, while its regular presence on decaying scales of coconuts—a substrate exceptionally rich in black yeasts but where *E. dermatitidis* remained absent—was remarkable. Babassu coconut scales are rich in fatty acids and etheric oils; the relative abundance of lipase genes in *E. spinifera* is consistent with this habitat choice. *E. dermatitidis* is highly selected by hot and moist indoor facilities, particularly steam baths and dishwashers, where temperatures periodically are 60–90°C. *Exophiala spinifera* has not been observed in domestic environments, which not only indicates differences in ecological preference, but also in exposition to human hosts, e.g., inhalation by patients with cystic fibrosis.

In general *E. spinifera* was physiologically more active, as judged from its response to proteins, lipids, ureum, and acid production. The repertoire of protease families in *E. spinifera* and *E. dermatitidis* according to MEROPS classification (Teixeira et al., 2017) revealed striking protease family expansions in *E. spinifera*, such as the families M38, S09X and S33. These expansions are absent in *E. dermatitidis* and might be responsible for the differences in the protein degradation profile. Abundance of proteases at the expense of carbohydrate-active enzymes is often taken as an indication of vertebrate pathogenicity (El Kaoutari et al., 2013). *Exophiala spinifera* possesses multiple copies of genes involved in α-glucan metabolism, which is considered an essential virulence factor in chromoblastomycosis (Teixeira et al., 2017). Nonetheless the LD50 of *E. spinifera* was comparable to that of *E. dermatitidis* in the *Galleria* model, indicating no obvious difference in virulence between the two species. However, when larvae were infected with *E. spinifera* the time-to-death was prolonged compared to larvae infected with *E. dermatitidis*, indicating that at least in *G. mellonella* larvae *E. spinifera* was more virulent. Furthermore, the origin of the isolates appeared also to be important. In both *E. dermatitidis* and *E. spinifera*, a shorter time-to-death was obtained with clinical isolates compared to environmental isolates.

The species also showed more tolerance to cycloheximide and hydrogen peroxide. The response of both species to elevated oxygenic action was mostly by yeast conversion (Figure 7, Table 3) and loss of melanin (Figure 6, Table 8) rather than by the formation of muriform cells and melanization. This response is not in line with the expected pattern in agents of chromoblastomycosis. We therefore consider published cases of human chromoblastomycosis by *E. dermatitidis* or *E. spinifera* (Rajam et al., 1958; Li et al., 2011; Lanternier et al., 2015; Srinivas et al., 2016) as questionable. *E. dermatitidis* shows a higher degree of thermotolerance, as expressed in its prevalence in hot indoor wet cells. Temperatures in steam baths and dishwashers intermittently are far above the permissive temperature for growth. Tesei et al. (2015) demonstrated that the fungus upon hostile conditions turns down its metabolism rather than showing a physiological stress response, which might be a survival mechanism for the super-extreme. It is speculated that *E. spinifera* lacks this strategy, as it has never been isolated from indoor habitats with extreme temperatures.

In *E. spinifera*, two patient populations have been described, i.e., healthy children and adolescents on the one hand, and elderly patients on the other. Somewhat unexpectedly, the former group was associated with fatal disseminated infection, whereas the latter only developed mild disease with successful cure. In contrast, dissemination in *E. dermatitidis* mostly occurred in East-Asian immunocompetent patients, and involvement of cervical lymph nodes and central nervous system was frequently reported. The species has a global distribution in the domesticated environment (Sudhadham et al., 2008), and therefore it remains unexplained why this type of infection is nearly limited to East Asia.

In both species investigated, molecular diversity has been reported. *E. dermatitidis* has several ITS-based genotypes, but our study demonstrated that ribosomal variation did not correspond with variation in protein-coding genes, and thus the species can be regarded as a single biological entity, clearly separated from its nearest neighbor species *E. phaeomuriformis*. *E. spinifera* showed a nearly identical degree of intraspecific variation; only the distinction of a small cluster known as *E. exophialae* was supported in all partitions, confirming this group as a neighboring species. This led to the conclusion that *E. dermatitidis* and *E. spinifera* cannot be meaningfully subdivided, and no lineages with reduced gene flow seem to exist. The found degrees of variation in barcoding genes (ITS Hd = 0.76 and 0.71 in *E. spinifera* and *E. dermatitidis*, respectively) may be taken as a model for black yeasts. No ascosporulating sexual states have been observed in either of them—although non-sporulating fruiting bodies have been reported (Gueidan et al., 2008) in *E. dermatitidis*, and in both species only a single mating type (*MAT1-2*) has been observed (Teixeira et al., 2017), thus possibly propagation and evolution is largely by independent clones. All data indicate that variation in both species is limited to the strain level, and no predictive grouping is possible.

Strain-level variation did not show significant correlation between clinical and environmental origins. Phenotypic intraspecific variation was found e.g., in protease, urease and acid production, but none of these parameters could be linked (Table 3). Antifungal susceptibility shows some variability in both species (Badali et al., 2012), and this could not be linked to other types of variation either. Given the rather significant variation in susceptibility, it is recommended always to perform *in vitro* prior to therapy.

It is safe to say that neither species behaves as a human pathogen, i.e., having enhanced fitness by the use of human vectors. Rather, human infection is extremely coincidental, not playing a role in the evolution of the fungi, and therefore typically opportunistic in nature. However, our data show that even related opportunists sharing essential virulence factors in yeast phases and capsular budding cells may be clinically very different. Surprisingly, the majority of cases of both species occurred in patients without known immune disorder, and the frequency of black yeast infection does not seem to increase with growing populations of compromised patients. Possibly several of the disseminated cases were patients with hidden congenital immune defects such as CARD9 deficiency (Lanternier et al., 2015). Idiopathic invasive fungal infections should lead to a search for underlying inborn errors of immunity (Alcais et al., 2010; Casanova and Abel, 2013; Lanternier et al., 2015), but also to different sources of exposition and windows of opportunity for microbial growth.

## Author contributions

YS carried out the literature search, strains collection, DNA extraction and sequencing, phylogenetic tree construction, physiology tests, Galleria mellonella virulence experiments, participated in the data analysis and drafted the manuscript. WL-vdS carried out the design of Galleria mellonella experiment and participated in the data analysis. LM carried out the genome data analysis and interpretation. BG participated in the data interpretation and analysis. RL and SdH participated in the design of the study, statistical analysis and manuscript revision and review. All authors read and approved the manuscript.

### Conflict of interest statement

The authors declare that the research was conducted in the absence of any commercial or financial relationships that could be construed as a potential conflict of interest. The reviewer JAMÁ and handling Editor declared their shared affiliation.
